# An investigation of feature reduction, transferability, and generalization in AWID datasets for secure Wi-Fi networks

**DOI:** 10.1371/journal.pone.0306747

**Published:** 2025-01-02

**Authors:** Nashmia Khalid, Sadaf Hina, Khurram Shabih Zaidi, Tarek Gaber, Lee Speakman, Zainab Noor

**Affiliations:** 1 Department of Computer Science, University of Engineering and Technology, Lahore, Pakistan; 2 Department of Computer Science, School of Science, Engineering & Environment, University of Salford, Salford, United Kingdom; 3 Oncampus UK North, University of Central Lancashire, Preston, United Kingdom; 4 Department of Computer Engineering, University of Engineering and Technology, Lahore, Pakistan; University of Central Punjab, PAKISTAN

## Abstract

The widespread use of wireless networks to transfer an enormous amount of sensitive information has caused a plethora of vulnerabilities and privacy issues. The management frames, particularly authentication and association frames, are vulnerable to cyberattacks and it is a significant concern. Existing research in Wi-Fi attack detection focused on obtaining high detection accuracy while neglecting modern traffic and attack scenarios such as key reinstallation or unauthorized decryption attacks. This study proposed a novel approach using the AWID 3 dataset for cyberattack detection. The retained features were analyzed to assess their transferability, creating a lightweight and cost-effective model. A decision tree with a recursive feature elimination method was implemented for the extraction of the reduced features subset, and an additional feature wlan_radio.signal_dbm was used in combination with the extracted feature subset. Several deep learning and machine learning models were implemented, where DT and CNN achieved promising classification results. Further, feature transferability and generalizability were evaluated, and their detection performance was analyzed across different network versions where CNN outperformed other classification models. The practical implications of this research are crucial for the secure automation of wireless intrusion detection frameworks and tools in personal and enterprise paradigms.

## 1. Introduction

Wireless transmission networks have led to substantial advances in data networking and communications, as well as the establishment of integrated networks. The rapid progress of information and communication technologies (ICTs) has offered numerous benefits to system users, but these technologies also have various vulnerabilities that might be exploited by network adversaries [1]. Cyberattacks such as malware attacks, classified data breaches, denial of service, phishing, and other security-related incidents have increased significantly in recent years. A cyberattack or a cyber threat refers to any unauthorized event or trespassing that compromises the network and carries out diverse malicious operations such as identity theft, spoofing, exfiltration, or exploitation of sensitive data and network resources [2]. A cyber-attack identification mechanism is a proactive approach that analyzes network traffic, identifies anomalies, and classifies cyber threats in the network [3].

Wi-Fi, or the IEEE-802.11 wireless local area networking (WLAN) standard, is crucial in daily life. IEEE-802.11 networks are at the forefront of this rapid change to a wireless space due to their potential to provide fast speed, enhanced mobility, usability, and cost-effective installation and maintenance expenses [4]. IEEE802.11-based wireless networks are widely used in homes, businesses, and public places, but also in critical infrastructures such as hospitals or manufacturing facilities where their availability is vital. Wi-Fi’s success may be attributed to a variety of factors, including well-defined use cases, deployment and configuration flexibility, and the accessibility of inexpensive, highly interoperable hardware [5].

As IEEE802.11-based networks became more ubiquitous, so did the possibility for hackers and other malicious activities to exploit them. Wi-Fi networks were initially open with data moving over the unencrypted medium. Individuals connected to their companies through public Wi-Fi networks such as coffee shops or libraries were always vulnerable to security threats. Anyone with a Wi-Fi receiver in the public space premises could access and interpret the sniffed data. Over the years, several approaches have been introduced to prevent security threats. Wired equivalent privacy (WEP) was the first scheme for the prevention of cyberattacks, but it had several flaws and soon became unreliable [6]. Later, Wi-Fi-protected access (WPA), WPA2, and WPA3 were introduced to secure wireless networks via authentication and encryption [7]. However, these standards are also vulnerable to cyberattacks with compromised encryption keys as authentication/association attacks are risky if the pre-shared key is down even though protected management frames (PMF) are operative.

Conventional or traditional intrusion detection systems require skilled human expertise to analyze network traffic patterns for cyber-attack detection, and attackers are generally familiar with the working of these mechanisms [8–10], which leads to several challenges. With advancements in network environments and the use of transformative technologies, the nature of attacks is also modified. Therefore, contemporary intrusion detection systems leverage advanced technologies such as machine learning or deep learning for cyber-attack detection in a specific environment including Wi-Fi in an IoT environment The amount of network data has significantly risen due to the increasing prevalence of connectivity, cloud services, and the Internet of Things (IoT) [11]. Due to this huge volume of data transmission through modern high-speed/ bandwidth communication networks, cyber-attack detection has become inefficient [12]. Due to this, in-depth automated monitoring of network traffic is required to identify distinct network attacks. Machine learning and deep learning approaches have the potential to revolutionize technology and operations as they address the problem of big data. Neural networks and various other deep learning techniques consistently achieve commendable results in addressing classification problems [13]. Incorporating these techniques allows for intelligently analyzing and discovering useful insights and patterns to detect attacks or security threats [14]. This could be the key to lightweight and cost-effective intrusion detection systems. This domain’s main shortcoming is that most publicly available benchmark wireless traffic datasets are outdated and do not include recent attack scenarios such as key reinstallation (Krack) or unauthorized decryption (Kr00k) attacks. It is crucial to acknowledge that the AWID3 dataset stands out as an exception in this regard, as it encompasses a more comprehensive range of scenarios, including those involving Krack and Kr00k attacks. Therefore, it is imperative to highlight the significance of the AWID3 dataset, emphasizing its relevance. Existing wireless attack datasets do not include the enterprise version of the 802.11 protocols. Another essential overlooked factor is the selection of appropriate performance metrics as accuracy measures do not demonstrate insights into the results [15–17]. However, it’s important to note that this specific issue falls outside the scope of the current research. Therefore, an effective system is required that can indicate any data breach or vulnerability in the network before any major loss of sensitive data.

Additionally, cyberattack detection is still a major challenge due to the ubiquity of successful cyberattacks publicized in the mainstream media. While there are some incredible cyber-attack detection results, they frequently rely on certain datasets and can’t always work well in a variety of real-world settings. In other words, while these models can thrive on their training data, their performance on varied network traffic remains uncertain. This highlights the need for intrusion detection features that are successfully transferable across different 802.11 datasets. The concept of feature transferability is especially significant when obtaining labeled data for the first time is excessively expensive, time-consuming, or unattainable. These are the potential features that continuously perform well across various scenarios. Transferable features, in the context of deep learning or machine learning models, are those that demonstrate consistent performance and efficacy across a range of datasets or scenarios [18]. If the features consistently maintain their performance well across diverse datasets, it shows that the proposed cyber-attack detection model has real-world application potential under a variety of network environments. Conversely, if the transferability of the features is limited, it will prompt further investigations to refine the feature selection process or develop more flexible models for broader applications in different network environments.

While extensive research has been conducted to improve the security of Wi-Fi networks, a distinct focus on Krack and Kr00k attacks appears to be lacking. The Krack vulnerability exploits instil flaws in the 4-way handshake protocol, allowing an attacker to reinstall a key that is momentarily in use. This, in turn, could end up in the decryption of Wi-Fi traffic, allowing unauthorized parties to discreetly intercept important information. Conversely, the kr00k attack occurs when a device disconnects from a Wi-Fi network while still encrypting data. Kr00k exploits a weakness in this circumstance by manipulating the flow of unencrypted packets, revealing fragments of previously encrypted data. Given the rapid growth of cyber threats, this omission creates a crucial information gap in the attempts to adequately protect wireless networks. Additionally, it’s evident that a substantial portion of prior research heavily relies on the AWID dataset. This dataset, however, has shown limitations over time, particularly because it does not include the most recent attack instances. This disparity is especially obvious in the case of protected management frames (PMF), a critical component in modern secure Wi-Fi networks. The absence of PMF in AWID is an important consideration for evaluating intrusion detection systems in the context of modern Wi-Fi security because it plays a critical role in reinforcing the authentication and association process. Another shortcoming is that many of the previous studies have focused on home-based Wi-Fi environments. These studies failed to recognize the necessity of testing their techniques and solutions in enterprise network environments. As the network setups, protocols, and security need to change significantly in corporate settings, this omission limits the practical relevance of research findings. Additionally, the absence of evaluation of generalization and transferability of features, so that the features can be used across different network conditions, is a major shortcoming in the existing literature. In this study, an innovative, lightweight cyber-attack detection model is proposed to identify existing attacks. These include Krack, Kr00k, de-authentication, and disassociation attacks. In the proposed methodology, recursive feature elimination (RFE) was used to extract 8 out of 16 MAC layer and physical layer features, proposed by [4], and tested using several classifiers including decision trees (DT), random forest (RF), extra trees (ETs), light gradient boost machine (GBM), multi-layer perceptron (MLP) and convolutional neural network (CNN). Moreover, the extracted features were used for the analysis across different datasets to test whether the given features are conceivably transferable. The results of this research offered valuable information regarding how transferable and generalizable the retained features are. If the features consistently show effective performance across diverse datasets, it suggests that the proposed cyber-attack detection model can be successfully implemented in real-world scenarios with varying network conditions, making it more practical and valuable. The following are the main contributions of this work:

- A decision tree with recursive feature elimination has been used to extract a reduced feature set. Several classifiers were tested on these features for attack detection.
- The transferability of the extracted features has been evaluated with AWID and AWID 3.
- A decision tree with RFE was used to extract a reduced feature set of the most meaningful features for each attack. These features expedite the attack detection process with a reduced number of computations and training time.
- Feature generalization of these reduced feature sets has been studied across the different data sets. Selected features for de-authentication and disassociation attacks from AWID 3 have been used in the AWID dataset for classification.

The summary of this research is structured in the following manner: section 2 sheds light on existing literature regarding cyber-attack detection. Section 3 discusses the pre-processing and feature selection process. Tree-based and MLP approaches for cyber-attack detection are reviewed in Section 4. Section 5 presents experimentation and results including feature transferability. Research work is concluded in section 6, with future work.

## 2. Literature survey

The three primary methods for analyzing network traffic to detect attacks are classified as signature detection, anomaly detection, and hybrid techniques that integrate both signature and anomaly detection techniques [19]. Signature-based detection identifies cyberattacks using predetermined signatures stored in the signature database. Whenever an attack occurs, the attack’s signatures are compared with the signature database, and the alert is generated if the attack signatures match the ones in the database. The signature database needs to be updated constantly to keep up with new attacks. Still, this technique only detects those attacks that are present in the database and does not detect zero-day attacks [20–22]. Anomaly detection is a dynamic approach that analyzes network traffic and notifies if there is any anomalous variation or abnormal behaviour in the network. Although it detects unknown attacks, there exists a greater risk of a high false-positive rate (FPR) as not every anomaly or variation in the network is a sign of intrusion [8, 23]. Conventional intrusion detection technology has been extensively studied for the past few years. The integration of AI, however, has transformed it even if it might not have excellent real-time detection performance. Nevertheless, researchers are focusing on machine learning (ML) and deep learning (DL) techniques since they have demonstrated a considerable increase in accuracy and a reduction in FPR. Several widely used publicly available benchmark datasets including NSL-KDD, CIC-IDS2017, AWID, and UNSW_NB15 are available for research purposes.

### 2.1. Conventional network intrusion research paradigm

Various ML and DL approaches have been proposed which can improve efficiency and lessen the execution time of intrusion detection mechanisms. In a research work [24], multiple supervised learning techniques embracing artificial neural network (ANN), decision tree, random forest, and unsupervised techniques including K-means, self-organizing map (SOM), and expectation maximization (EM) algorithms were applied to CIC-IDS2017. Some algorithms demonstrated high accuracy while others such as SOM and EM failed to detect targeted attacks. A novel network structure of deep belief network (DBN) was proposed based on an artificial fish swarm algorithm (AFSA), genetic algorithm (GA), and particle swarm optimization (PSO) to detect network intrusions in NSL-KDD [25]. Although this model attained 98% accuracy, a higher number of layers can increase computational costs. The work in [26] proposed a hybrid technique to detect intrusions based on feature selection and classification using UNB ISCX 2012 and CIC-IDS2018 datasets in the Apache Spark environment. A stacked auto-encoder (SAE) performed feature selection and a support vector machine (SVM) algorithm was used for intrusion detection. Results demonstrated 90.2% accuracy with reduced training time. A hybrid technique consisting of K-means clustering with RF, CNN, and long short-term memory (LSTM) was applied in the Apache Spark environment [27]. Adaptive synthetic sampling was used to solve imbalanced datasets. The results showed 85% accuracy on NSL-KDD and 99.9% accuracy on CIC-IDS 2017. In [28], principal component analysis (PCA) and mutual information (MI) with LSTM were implemented for dimensionality reduction and classification of cyber-attacks. LSTM-PCA achieved the highest accuracy of 99.36%. Three feature selection techniques comprising autoencoder (AE), the stacked autoencoder (SAE), and deep autoencoder (DAE) with DNN were applied to indicate data breach in CIC-IDS2018 and NSL-KDD [29] where DAE-DNN attained the highest accuracy. DAE for feature selection and recurrent neural networks (RNN) for classification were implemented on CIC-IDS2018 and Bot-IoT [30]. The highest accuracy for the Bot-IoT dataset 98.39% was obtained with DAE while significant results for CIC-IDS2018 were obtained with RNN, 97.38% accuracy. The major shortcoming was the lack of details of actual experimentation. In this work [32], The BAT optimal feature selection method to identify the most relevant features. To evaluate the accuracy of intrusion detection, the Support Vector Machine (SVM) classifier was tested using the KDD99 benchmark dataset. When compared to alternative machine learning algorithms, this approach outperformed others with a detection accuracy of 99%. The Perceptual-Pigeon-Galvanized-Optimization(PPGO) approach was used to choose the best parameters for intrusion detection in datasets NSL-KDD, CICIDS, and Bot-IoT [33]. Then the Likelihood Naive Bayes (LNB) classification method was implemented outperforming previous models with a remarkable accuracy rate of 99%. The study introduced a novel feature selection method based on the Capuchin-Search-Algorithm (CapSA). CNN-CapSA was evaluated using four IoT-Cloud datasets: NSL-KDD, BoT-IoT, KDD99, and CIC2017, and surpassed other state-of-the-art methods with approximately 99% accuracy. The study [34] proposed HetIoT-CNN IDS, an advanced Intrusion Detection System (IDS) that used a Convolutional-Neural-Network (CNN) built for the HetIoT (Heterogeneous Internet of Things) environment. The HetIoT-CNN IDS achieved high accuracy scores of 99.75% for binary classifications, 99.95% for 8-class classifications, and 99.99% for 13-class classifications.

### 2.2. Contemporary Wi-Fi intrusion research paradigm

The significance of intrusion detection in securing networks has drawn the attention of numerous researchers. Numerous publications proposed novel methodologies for intrusion detection for wireless sensors and Wi-Fi networks. Technologies like wireless networks, 4G, IoT, and others transmit a substantial amount of data and are pre-disposed to various cyberattacks and security risks that might jeopardize the reliability and confidentiality of information or services. Wi-Fi networks are nearly universally used in businesses nowadays to give employees access to the Internet. Business stakeholders have become more concerned about Wi-Fi networks and operational security. As the dynamics of technology and attack strategies are expanding, the IDS must be scalable and adaptable to counter new attacks.

Several techniques have been proposed to detect cyberattacks on wireless networks. In [34], two models were introduced to draw out additional features using SAE, the features were then combined with the original features based on the amount of mutual information shared between the features and class labels. It was then merged with the radial basis function classifier (RBFC) to evaluate results on the AWID dataset. Results showed that RBFC acquired 98% accuracy with 7 optimal features. A novel system KTRACKER was proposed to detect novel cyber threats such as key re-installation (Krack) on Wi-Fi-protected access (WPA2) [35]. It grouped handshake packets and used traffic analysis to find KRACK. Cat boost attained the highest accuracy out of the three machine learning models XGBoost, Light Gradient Boosting Machine (Light GBM), and Cat boost. In a recent study, a feed-forward-deep-neural-network (FFDNN) wireless-IDS system using a wrapper-based feature-extraction unit (WFEU) was introduced [36]. The WFEU extraction approach involved the extra trees algorithm to extract optimum feature selection. The proficiency of the proposed model was examined using the UNSW-NB15 and AWID intrusion detection datasets. The proposed model acquired higher detection accuracy than existing techniques. Overall, the accuracies of 99.66% and 99.77% with 26 features from AWID, and 87% and 77% with 22 features using UNSW_NB for binary and multiclass classification were attained respectively.

A novel, conditional deep-belief-network (CDBN), technique was proposed to detect wireless network intrusions in real-time and identify cyber-attacks [37]. A stacked contractive auto-encoder (SCAE) approach was presented for the reduction of data dimensionality to mitigate the effects of its unbalanced nature and data redundancy on detection accuracy. The experimental results showed better detection accuracy and speed, with an average detection time of 1.14 ms and 97.4% detection accuracy. Most modern IDSs utilize machine learning approaches that suffer from performance deterioration when used against an adversary and are unable to achieve a balance between accuracy and false-positive rate (FPR). Due to the open-sharing nature of wireless technology, organizations continue to have serious concerns about Wi-Fi security. A significant number of impersonation attacks were misclassified into injection attacks in previous studies. To overcome this limitation, a dual-stage Wi-Fi network-intrusion-detection (WNIDS) method, based on machine learning, was proposed to increase the detection accuracy for injection and impersonation threats in a Wi-Fi network [38]. In the first stage, the RF outperformed other models to classify the attacks into three classes normal, flooding, and unified impersonation or injection attacks from the AWID-CLS-R test dataset. In the second stage, NB outperformed other models by correctly classifying the unified attack instances into impersonation attacks and injection attacks with an accuracy of 99.42%. To prevent overfitting, a feature separation approach based on word embedding was developed to speed up calculations [39]. For classification, a dual/limited attention mechanism was proposed instead of global attention. These approaches were utilized with the UNSW-NB15 and AWID datasets where the gated recurrent unit (GRU) attained the highest accuracy of 93.47% on the AWID dataset and RNN attained 94.96% accuracy on the UNSW-NB15 dataset. However, only the accuracy metric was used as an evaluation metric even though accuracy alone is not a reliable metric.

Another novel system, the Wi-Fi intrusion-detection-system (WIDS), proposed an anomalous behaviour analysis technique to identify assaults on Wi-Fi networks with significantly high accuracy and reduced rate of false alarms [40]. In this technique, n-grams were implemented to represent the normal behaviour of the Wi-Fi protocol, and several machine learning models were used to distinguish Wi-Fi traffic as normal or malicious. This technique was evaluated using numerous datasets gathered locally at the University of Arizona and the AWID dataset classified all Wi-Fi protocol assaults with low false positives (0.0174) and a variable low rate of false negatives for different attacks. [41] classified DoS attacks using an ensemble technique. Recursive feature elimination (RFE) was used for the selection of features and then an ensemble classifier, using RF, SVM, and Swell with 10-fold cross-validation, for classification with AWID-CLS-Test dataset. The outcomes demonstrated a precision of 99.98% and 0.12 FPR. For wireless intrusion detection, a feature selection technique based on Fuzzy C-Means (FCM) was introduced, which used the distance between the FCM centre point and the data point to determine the difference between the normal and attack centre distances, and then used the distances to pick the features [42]. This method was tested using the AWID dataset, and the findings demonstrated that it was quite accurate in attack detection. Researchers have lately deemed the 5G network environment to be significant, owing to the advancement of network communication and the growing number of users. As a result, wireless network security of 5G networks has become a crucial concern. This study made two major advances in the detection of network assaults [43]. Numerous ML and DL approaches, including multi-class neural networks, multi-class decision jungle, decision trees, KNN and multi-class decision forest were used to construct an intelligent system that classifies data into normal and abnormal traffic to detect cyber assaults. Using the AWID3 dataset, the performance was evaluated using the Omnet++ simulator tool to retrieve a subset of the packet transmission performance dataset for a run time of 20 seconds. This network attained 99% accuracy, however, only accuracy is used for evaluation metrics. Furthermore, ‘frame.time.epoch’is a time series feature and should be preprocessed accordingly. [44] proposed an intrusion detection technique for wireless sensor networks based on graph neural networks and Lyapunov optimization in this study. The AWID dataset was utilized for GNN with the Lyapunov optimization loss function. The acquired results were better than the previous SVM-based works. However, no confusion matrix or false alarm rate has been calculated. By resampling training data and redefining rewards in reinforcement learning, the research creates an environmental agent that improves intrusion detection [45]. In a multi-classification experiment, the system, AE-SAC, achieves excellent performance, with an accuracy of 84.15% and an F1-score of 83.97% on the NSL-KDD dataset and an accuracy and F1-score exceeding 98.9% on the AWID dataset. Related work with the critical analysis is presented in Table 1.

**Table 1:** Significant summary of literature.

Title	Problem	Feature selection	Technique	Dataset	Result	Gap
Wei et al. [25] - 2019	Optimization method for IDS	N/A (41)	PSO- AFSA-GA-DBN	NSL-KDD	99.85%	Doesn’t reflect modern traffic scenarios
Kasongo et al. [36]—(2020)	Feature reduction for wireless IDS	WFEU	FFDNN	UNSW_NB15, AWID	77.17%, 99.77%	Absence of multiclass classification
Reyes et al. [38]– 2020	Two-stage wireless IDS	RFE, Chi-square, Correlation, Feature Importance, PSO	RF, NB, SHAP, ET, XGBoost, Bagging, LightGBM	AWID	99.99%	Doesn’t include the latest Wi-Fi attack scenarios
Li et al. [39] -2021	Feature separation method for IDS to improve accuracy	Word Embedding	RNN, LSTM, GRU	AWID, UNSW_NB15	94.96%, 93.47%	only accuracy for evaluation measures
Laghrissi et al. [28]- 2021	LSTM approach for IDS	PCA, MI	PCA-LSTM, LSTM-MI	KDD99	99.44%	Outdated dataset
Sharafaldin et al. [24] -2021	ML for anomaly-based IDS	N/A	CNN, RF, ANN, SOM, EM, k-NN	CIC-IDS2017	Approx. 99%	
Mujahid et al. [43]—(2022)	Wireless IDS for 5g networks	Pearson correlation	DT, NN, kNN, Decision Jungle, Decision Forest	AWID3	99%	Time-based feature ‘frame.time.epoch’ is not preprocessed properly
Agrawal et al. [35]- (2022)	Krack detection using ML	N/A	LightGBM, XGBoost, Catboost	AWID3	87.12%	accuracy should be increased
Shitharth et al. [32]-(2022)	Optimization method for IDS	Perceptual-Pigeon-Galvanized-Optimization(PPGO)	Likelihood Naïve Bayes	NSL-KDD, CICIDS, and Bot-IoT	99%	Doesn’t include the latest Wi-Fi attack scenarios
Shitharth et al. [31]-(2022)	Optimal feature selection method for IDS	BAT Algorithm	SVM	KDD99,	99%	Doesn’t reflect modern traffic scenarios

In the extant literature, most of the research studies did not include modern Wi-Fi traffic. Many studies were conducted using publicly available datasets including even outdated KDD99 and NSL-KDD datasets launched in 1999 and 2009 with 42 features that do not reflect modern attack scenarios [24]. Other datasets that are widely used in research do not include the latest Wi-Fi attack scenarios such as ISCX 2012 is based on emulated traffic with 82 features and does not reflect the effectiveness of a practical network environment. It is comprised of over 2 million traffic packets, and attacks represent 2% of the whole traffic [26]. UNSW-NB15 is based on a simulated network and consists of 49 features, 175,341 normal traffic, and 82,332 anomaly classes making it a highly imbalanced dataset. In 2017, CIC-IDS2017 was introduced, and later CIC-IDS2018. These datasets contain various recent cyberattacks, such as brute-force attacks on FTP and SSH servers, denial-of-service attacks(DoS), Heartbleed attacks, and other online attacks such as XSS, SQL injection, and brute-force attacks. These statistics include assaults that were absent from the earlier datasets, such as infiltration, botnets, and DDoS attacks. Another benefit of this dataset is that the normal traffic generated in this dataset is based on network protocols such as HTTP, HTTPS, FTP, SSH, E-mail, etc., which is closer to a real-time network environment than the previous datasets [24]. The major shortcoming of the research with these datasets is that they do not include Wi-Fi attack scenarios. All these datasets are based on a wired network. Aegean Wi-Fi Intrusion Detection Dataset (AWID) is the only benchmark dataset that consists of attacks related to wireless intrusion networks. It provides a freely accessible dataset of legitimate and malicious traffic directed against 802.11 networks. This is the first dataset that includes 802.11 attacks [46] but still does not include Krack and Kr00k attacks. The focus of this work is to extract the most meaningful features to have a secure Wi-Fi system. Wrapper approaches, such as RFE, use machine learning algorithms to regulate the performance of selected features and frequently outperform filter methods in terms of predictive accuracy [47].

Furthermore, the existing literature failed to extract and analyze the generalized features for each attack including Krack and Kr00k, and authentication attacks which include de-authentication and disassociation attacks. AWID3 benchmark dataset includes these attacks and focuses on enterprise adaptations of the protocol unit thus considered more challenging than AWID and providing greater security methods.

However, another significant shortcoming in current research is the lack of evaluation on the generalization of trained models with other datasets. The lack of evaluation in this regard raises uncertainties about the transferability and generalizability of the features and models. Without such evaluation, it remains unclear whether the proposed cyber-attack detection model will perform well and provide accurate results in real-world scenarios with varying network conditions. Consequently, as a result, additional research and testing are crucial to ascertain whether the retained features can be used successfully across various datasets, ensuring their dependability and usability in a wider spectrum of network environments.

## 3. Methodology

**Fig 1:**
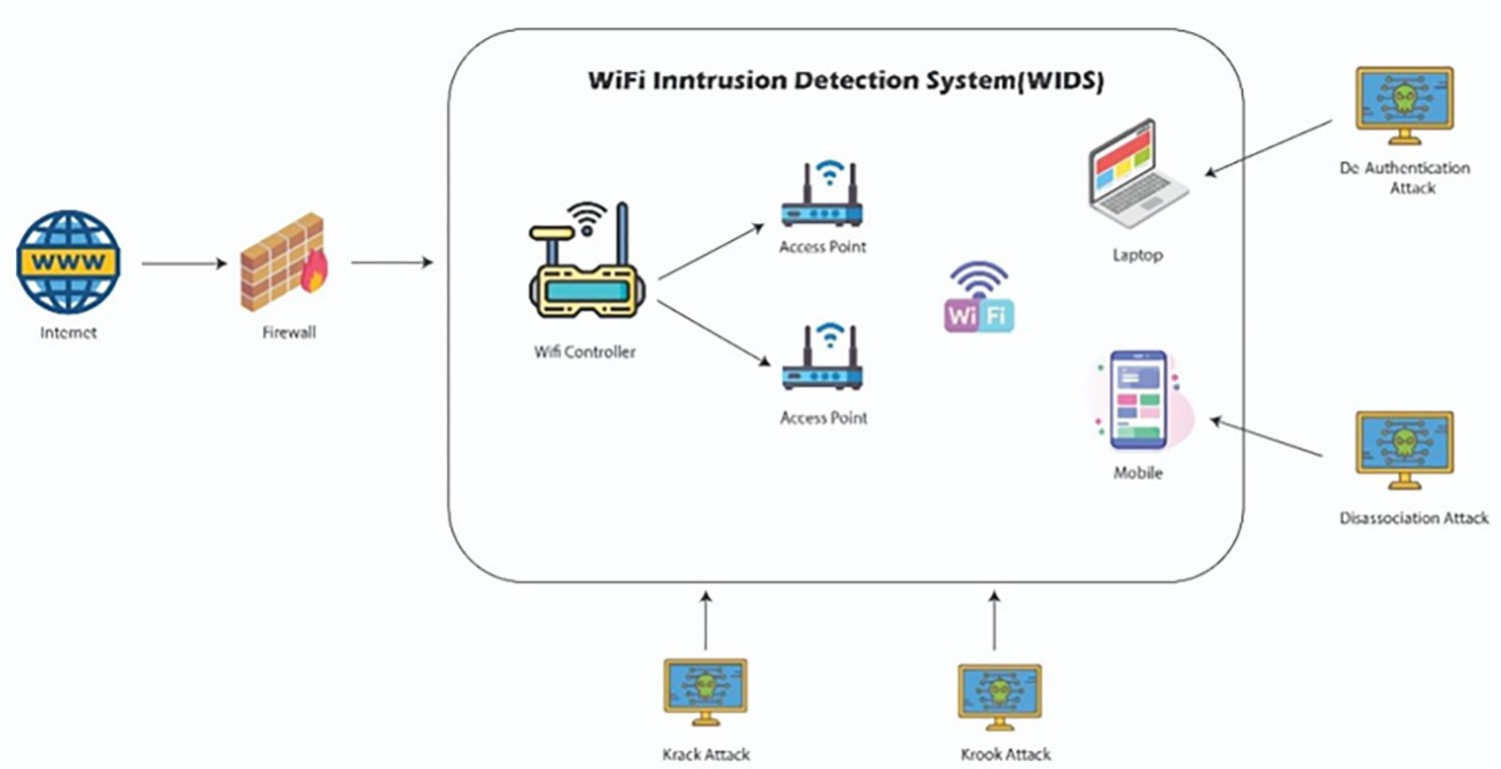
Wi-Fi intrusion detection system.

**Fig 2:**
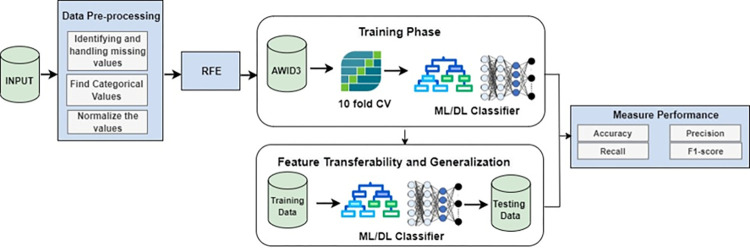
Proposed methodology.

Corporate Wi-Fi networks are vital for both businesses and public administrations, offering a highly adaptable and secure infrastructure. Access points face vulnerabilities like deauthentication, disassociation attacks, and the KRACK attack, which exploits the four-way handshake. Recently, the Kr00k attack has emerged as a critical threat, specifically targeting Wi-Fi chips. These risks demand vigilant security measures to protect wireless networks and devices. Fig 1 shows the framework for a secure Wi-Fi network. One of the prime objectives of this research is to improve the detection rate of attacks with fewer features. Fig 2 outlines the proposed methodology for this research study. To conduct the proposed strategy and experimentation, the AWID3 dataset is utilized. Generally, the datasets consist of missing values, special characters, and different data types. Therefore, preprocessing of the dataset is performed in the second step. In the third step, the feature selection algorithm is utilized to get a minimized set of features using a recursive feature elimination algorithm, and several classification algorithms including DT, RF, ET, Light GBM, MLP, and CNN were used for classification. In the next step, DT-RFE was used to get features for each attack and classification was performed to analyze the accuracy of these features. In the last step, it was analyzed if the features for each attack were transferable. It is worth noting that while deep learning algorithms are known for their capacity to automatically learn hierarchical features and could be powerful, they might come with increased computational demands. Deep learning models often require a large amount of data for training, and the effectiveness of such models is typically observed in more extensive datasets. As the AWID 3 dataset is relatively small and lacks the complexity that could benefit deep learning, simpler models like decision trees performed better.

### 3.1. Feature selection using recursive feature elimination

The purpose of feature selection is the minimization of the time and space complexity of the model. Detection of attacks through reduced features and processing time without any delay will lead to an efficient lightweight IDS. RFE is a method for feature selection that uses a classifier to build the model. A machine learning model is trained and assessed using various feature subsets to determine the optimal feature subset that leads to improved performance of the model. The RFE process begins with training a machine learning model on an entire set of features, followed by ranking the features in order of significance to the model’s performance. The model is then retrained and assessed with a smaller set of features after the least significant feature is eliminated. This process is repeated iteratively until a predetermined number of features is reached, or until a desired level of performance is achieved. The feature importance score for each feature is computed (Eq 1), and the feature with the lowest value is removed from the subset.


$$S(i)=\sum_{k=1}^{n}\left(r_{min}-r_{ik}\right)$$ Eq 1

Wrapper-based RFE differs from other feature selection methods, such as filter-based or embedded methods, in that it evaluates the impact of feature subsets on the specific machine learning model being used, rather than just measuring the correlation between features and the target variable.

### 3.2. Decision tree

The decision tree is a type of supervised learning technique to tackle classification problems. A DT’s components include leaves, branches, and nodes. The branches indicate the collection of features that result in the class labels, whereas the leaves represent the labels for each class. Both discrete and continuous data sets can be used with these branches. The samples are categorized into two or more homogeneous sets by the DT approach. The classification process works in a top-to-down sequence, and an optimal conclusion is attained when the proper category of the leaf node is discovered. However, decision trees face overfitting problems. Decision Trees separate data at each node using splitting criteria such as Gini impurity(D) from a different number of classes(C) in the dataset where p_i_ is the probability of an instance in D belonging to class i as shown in Eq 2.


$$Gini(D)=1-\sum_{i=1}^{C}({p_{i})}^{2}$$ Eq 2

For each feature and value, the algorithm examines the splitting criterion and chooses the one that minimizes the criterion. The data is split recursively into child nodes until a halting criterion is reached. It assigns a class label or numerical value based on the majority class or mean value when it reaches a leaf node. Algorithm 1 demonstrates the decision tree process.

**Algorithm 1:**
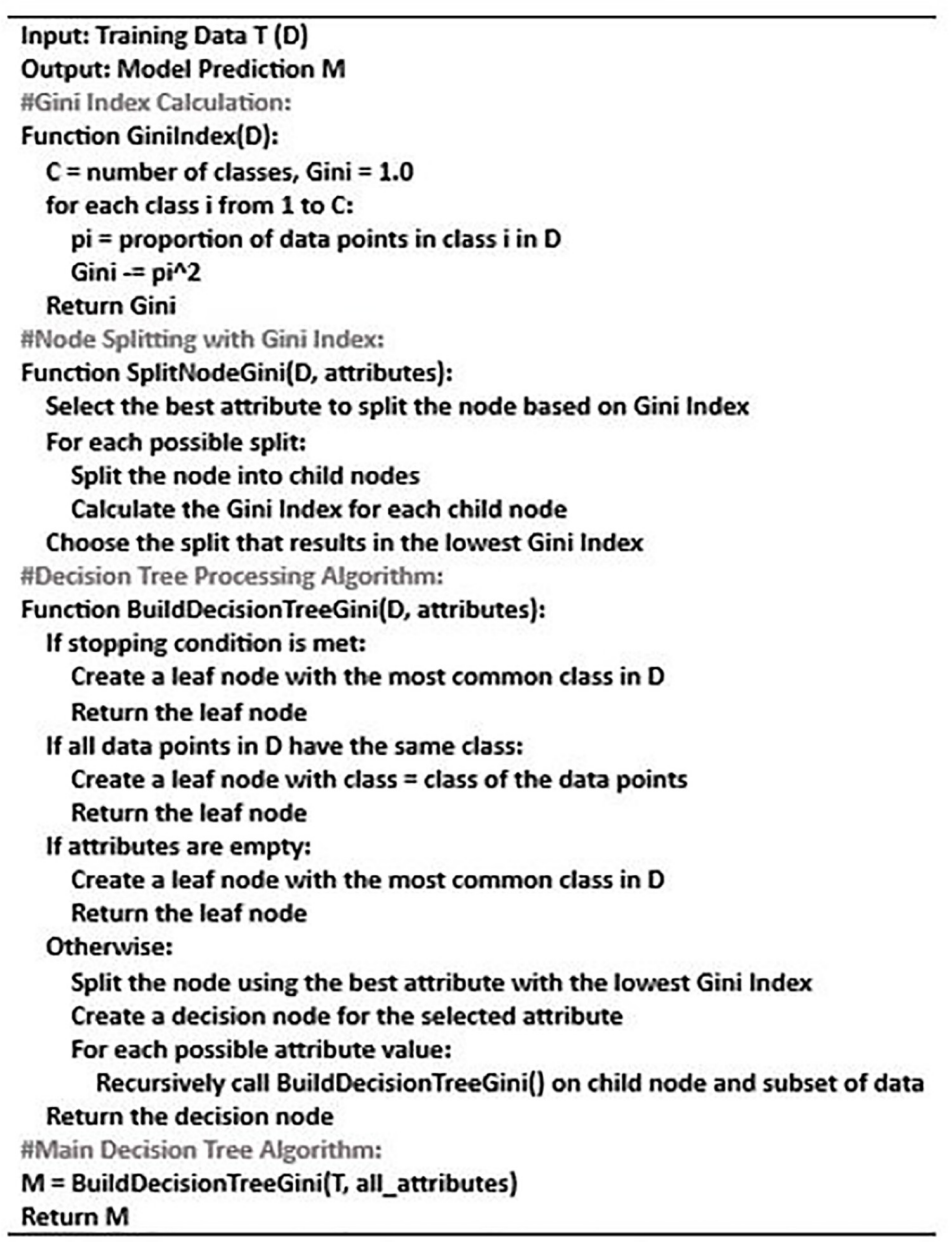
DT Algorithm.

### 3.3. Random Forest

The RF method, an ensemble learning technique, is used for tackling classification and regression issues. Unlike decision trees, the random forest classifier uses numerous DTs to classify a given dataset. These decision trees calculate the entropy of features and then split the samples layer by layer. As a result, the dataset instances are divided according to the desired column. Random forest overcomes the problem of overfitting as opposed to decision trees. Random Forest is an ensemble learning method that uses numerous decision trees to improve prediction accuracy. Let (X, Y) represent the dataset, with X representing the feature matrix of shape (n, p), with n instances and p features, and Y representing the target variable. Then bootstrap of the dataset is created by randomly picking n instances from the original dataset with replacement. The original dataset (Xb, Yb) is the same size as the sampled dataset (Xb, Yb). For each tree, choose a subset of m features at random from the total p features. Create a decision tree T with (Xb, Yb) and the randomly chosen m characteristics. Create N decision trees using the preceding procedure to construct a Random Forest {T1, T2,…, TN}. To determine the most prevalent class for classification, employ a majority vote among the tree predictions just like in Eq 3.


$$\hat{Y_{new}}=mode(T_{1}(X_{new}),T_{2}(X_{new}),\ldots ,T_{n}(X_{new}))$$ Eq 3

### 3.4. Extra Tree

This algorithm attempts to fit randomized decision trees on distinct sub-samples of the dataset and implements the notion of averaging to improve accuracy as well as efficiency to overcome the overfitting problem. The extra tree algorithm uses the standard top-down approach to generate a sequence of raw gradient or regression trees. ETs are distinct from conventional tree-based clustering algorithms in such a way that they separate nodes by arbitrarily cutting points and construct the tree using the entire learning sample. Extra trees are similar to random forests. Compared to Random Forest, Extra Trees can be trained more quickly because the split thresholds are chosen at random and there is no need to look for the best thresholds.

### 3.5. Light Gradient Boosting Machine

The LightGBM method incorporates two innovative techniques: gradient-based one-side sampling (GOSS) with exclusive feature bundling (EFB). XGBoost is a deep-learning algorithm used for regression and classification tasks. For classification, the goal is to minimize the log loss function for n number of data points where y_i_ is the true class label and p_i_ is the probability of class for data point I as given in Eq 4.


$$L(\theta )=\sum_{i=1}^{n}\left(y_{i}\log\left(p_{i}\right)+(1-y_{i})log(1-p_{i})\right)$$ Eq 4

It builds an ensemble of decision trees, called boosted trees, to make predictions. The objective function uses two regularization terms: L1 Regularization (Lasso) and L2 Regularization (Ridge), aiming to prevent overfitting and maximize gain scores. The ensemble’s predictions are weighted according to performance.

### 3.6. Multilayer Perceptron

One of the most often used feed-forward neural networks is the multi-layer-perceptron (MLP) neural network. MLP neurons are linked in a one-way and one-directional manner. The MLP design is as follows: the initial layer that feeds the network with input variables is called the input layer, the last layer is called the output layer, and all the layers in between are called hidden layers. The hidden layer, which consists of m neurons, computes a weighted sum of inputs and expresses it for the jth neuron by passing it through an activation function as shown in Eq 5. Where Z_j_ is the weighted sum of neuron j. The weight known as W_ij_ is what connects the ith input neuron to the jth hidden neuron and the bias for neuron j is b_j_. The weighted sum of the output neuron can be expressed as in Eq 6 where Z_k_ is the k-th output neuron’s weighted sum, c_k_ is the bias for the k-th output neuron, and V_jk_ is the weight connecting the j-th hidden neuron to the k-th output neuron.


$$Z_{j}=\sum_{i=1}^{n}(X_{i}.W_{ij})+b_{j}$$ Eq 5

$$Z_{k}=\sum_{j=1}^{m}(A_{j}.V_{jk})+c_{k}$$ Eq 6

It is provided with the necessary structural flexibility and representational capabilities, as well as access to a diverse set of data samples.

### 3.7. Convolutional Neural Network

CNNs are designed to learn spatial and temporal patterns in data. In the context of intrusion detection, CNNs can be used to learn patterns in the independent features of the dataset. The convolutional layer is the fundamental component of CNN, where a series of filters are applied to the input image to generate feature maps. The mathematical equation is given in Eq 7 where in the l^th^ feature map, Z_ij_^[l]^ is the value at position (i, j). The l-th layer’s filter at position (m,n) has assigned a weight W_m,n_^[l]^. In the (l-1)-th layer’s feature map, X_i+m, j+n_^[l-1]^ is the value at position (i+m, j+n). Following feature extraction, pooling layers are used to analyze the data and minimize the spatial dimensions of feature maps where P_i,j_^[l]^ is the pooled value at position (i, j) and A_2i2j_^[l],^ etc. are the values at corresponding positions in the activated feature map given in Eq 8. This is then followed by fully connected layers, which make the final prediction. The weights of the filters and fully connected layers are learned through training the network on a labeled dataset. This is demonstrated in algorithm 2. For example, if the independent features of the intrusion detection dataset are network packets, a CNN can be used to learn patterns in the packet’s header fields such as source-IP and destination-IP addresses, port numbers, and protocol type. By learning these patterns, CNN can detect anomalies in the network traffic, which may indicate an intrusion.


$$the\;Z_{i,j}^{\left[l\right]}=\sum_{m=0}^{fh-1}\sum_{n=0}^{fw-1}(W_{m,n}^{\left[l\right]}.X_{i+m,j+n}^{\left[l\right]})+b^{\left[l\right]}$$ Eq 7

$$P_{i,j}^{\left[l\right]}=\max\left(A_{2i,2j}^{\left[l\right]},A_{2i,2j+1}^{\left[l\right]},A_{2i+1,2j}^{\left[l\right]},A_{2i+1,2j+1}^{\left[l\right]}\right)$$ Eq 8

**Algorithm 2:**
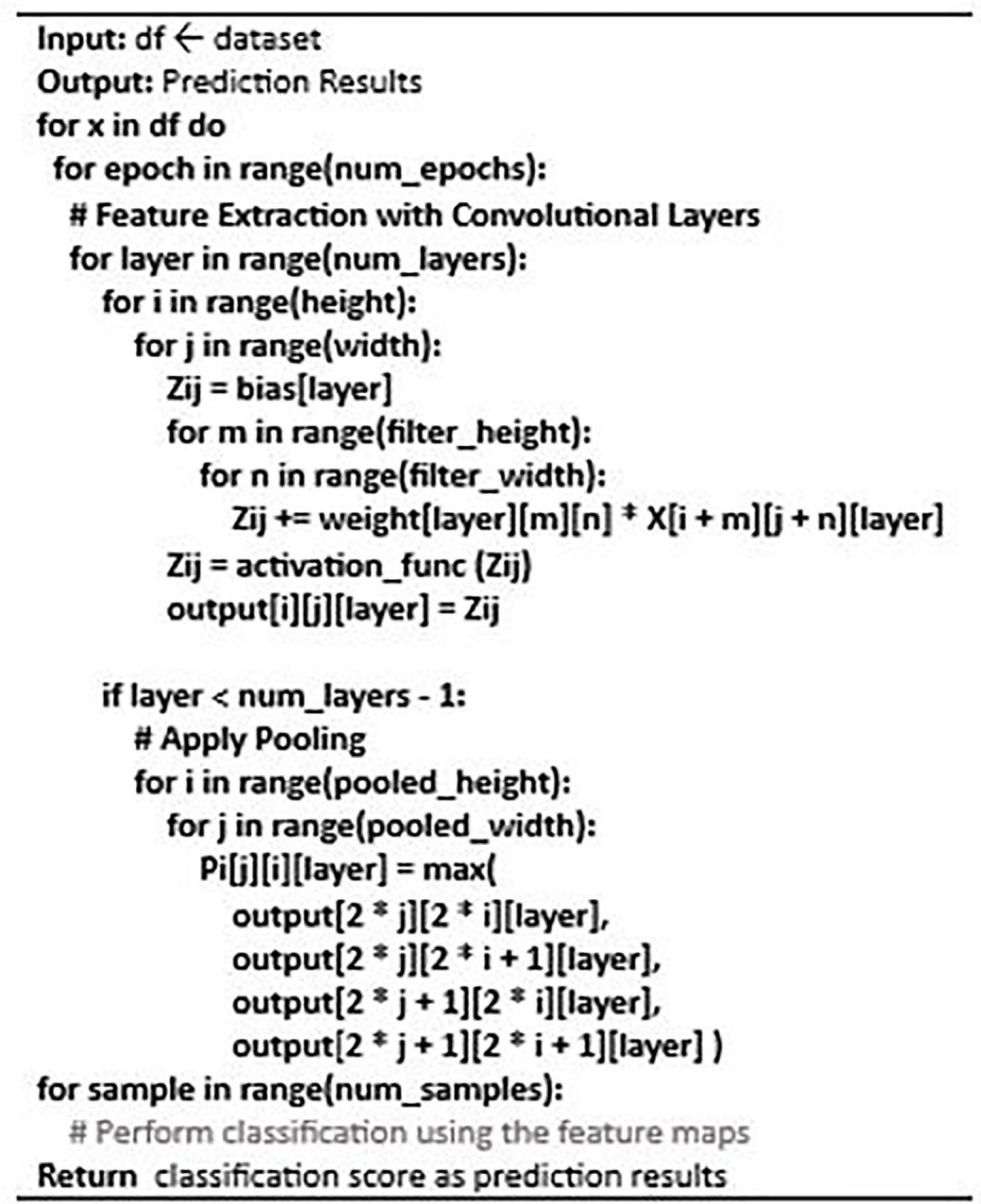
Convolutional Neural Network (CNN).

### 3.8. Feature transferability

The effectiveness of intrusion detection systems (IDS) is assessed using consolidated metrics such as precision, recall, F1 score, and AUC score. As anticipated, all IDS models achieve remarkably high F1 scores, ranging from 0.98 to 1, and AUC scores, ranging from 0.97 to 0.99, when trained and tested on individual datasets like AWID and AWID3. These findings are consistent with prior research on IDSs applied to publicly available datasets and underscore the models’ efficacy in their specific contexts.

However, the transferability of these high-performing models to an unseen dataset leads to diverse outcomes. The performance may vary, indicating that the models’ exceptional performance on a specific dataset does not automatically guarantee their ability to generalize to novel and unseen datasets under a distinctive network environment.

Undoubtedly, a pivotal question arises: Can the chosen set of features be seamlessly transferred across datasets? To probe this matter, the model, having undergone training with the retained features, undergoes comprehensive testing using unseen network traffic. This evaluation encompasses real-time scenarios and diverse network environments, empowering researchers to gauge the enduring efficacy and broad applicability of the retained features beyond the confines of the original dataset. This evaluation assumes paramount significance as it validates the feasibility and versatility of the proposed cyber-attack detection model in dynamic and varied network conditions.

### 3.9. Dataset description

Contrary to the first Aegean Wi-Fi Intrusion Detection Dataset (AWID), the AWID3 dataset focuses on enterprise adaptations of the protocol unit and is thus considered more challenging than AWID providing greater security methods such as the usage of protected management frames (PMF), introduced with the 802.11w amendment, and support for various network designs. AWID3 is a publicly accessible database of Wi-Fi network traffic that includes actual traces of both legitimate and unwanted 802.11 activity. It captures numerous different attacks launched against the IEEE 802.1X extensible authentication protocol (EAP) system. This dataset focuses primarily on attacks related to 802.11 and higher-layer attacks. Furthermore, new 802.11-specific attacks, such as Krack and Kr00k, have been included for analysis. In this research, a minimized edition of the dataset has been used consisting of four types of attacks de-authentication, dissociation, Krack, Kr00k, and benign traffic.

### 3.10. Data preprocessing

**Fig 3:**
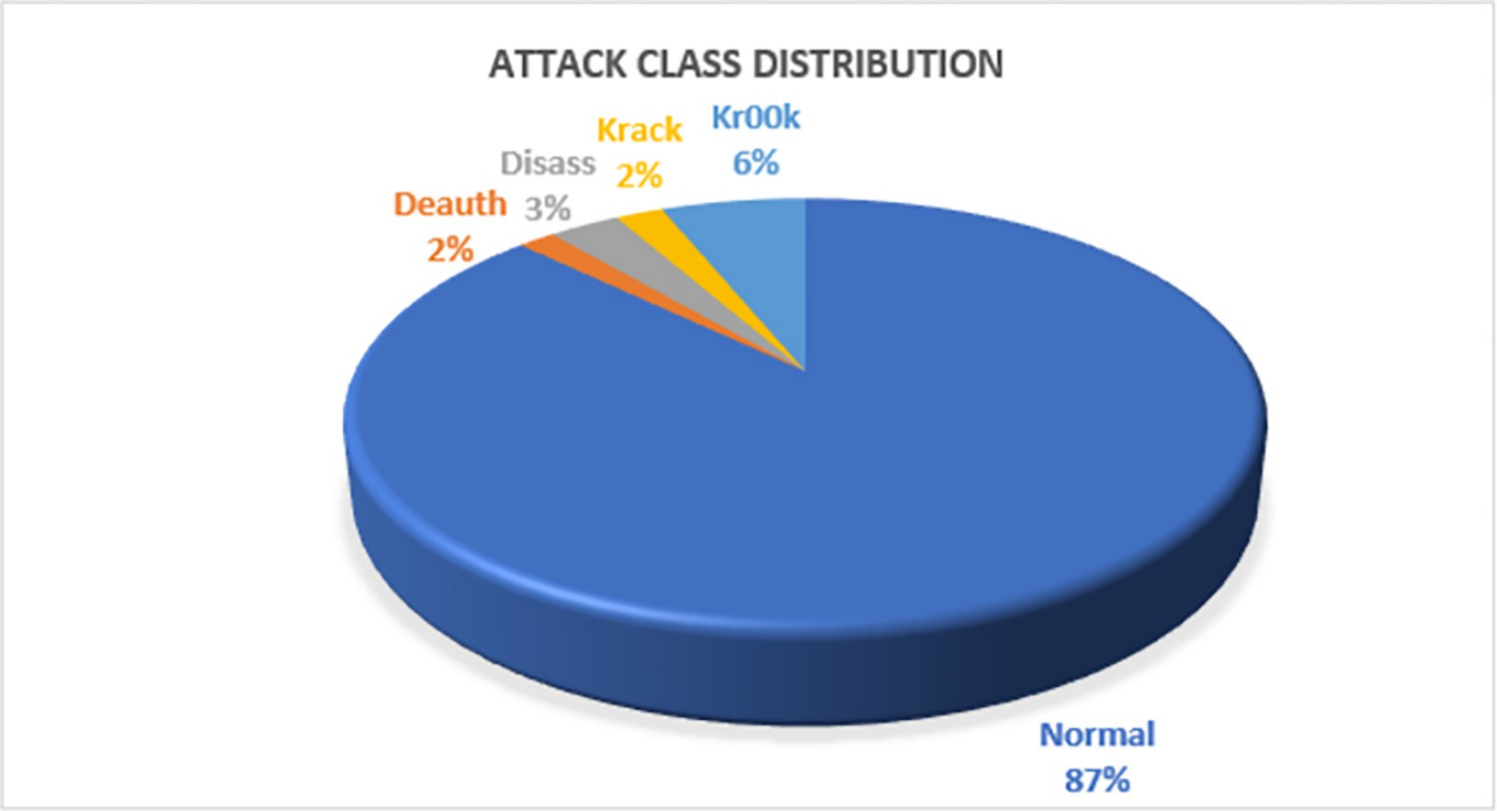
Classes distribution.

Timestamps, numerals, hexadecimal digits, strings, etc. are examples of features’ data types. The AWID3 dataset is an unbalanced distribution of records. For this research, the imbalance property of the dataset is not altered. Fig 3 demonstrates the distribution of the number of instances in the dataset.

For the feature selection phase, time-based features such as the frame.timedelta, frame.time_delta_displayed etc. have been discarded since the focus is not the time-based analysis. Only, cherry-picked MAC and physical layer features were selected and proposed in [4]. These features were considered due to their potential to function as a solid foundation for the development of a reliable, easy-to-handle, and economical 802.11 cyber threat detection system. The features with their description are presented in Table 2.

**Table 2:** Selected features.

Feature Name	Description
1. Frame.len	Frame Length
2. Radiotap.length	Frame header length
3. Radiotap.dbm_ansignal	Present flag antenna signal (dbm)
4. Wlan.duration	Duration time
5. Radiotap.present.tsft	Present flag (Timing Synchronization Function Timer)
6. Radiotap.channel.freq	Channel frequency value
7. Radiotap.channel.type.cck	Complementary Code Keying-flag
8. Radiotap.channel.type.ofdm	Orthogonal Frequency Multiplexing-flag
9. Wlan.fc.type	Type- Flag
10. Wlan.fc.subtype	Subtype-Flag
11. Wlan.fc.ds	Distribution System-status flag
12. Wlan.fc.frag	More fragments-flag
13. Wlan.fc.retry	Retry-flag
14. Wlan.fc.pwrmgt	Power management–flag
15. Wlan.fc.moredata	More data-flag
16. Wlan.fc.protected	Protected frame-flag

AWID3 dataset contains a few missing values in the instances. To encounter this problem, records that contain any missing values have been dropped. Here, wlan.fc.ds represents hexadecimal strings which are converted to numerical using label encoding. There exists a significant difference between the ranges of feature values, such as radiotap. channel.freq begins from 1000 while, the maximum value of radiotap. length for this dataset is approximately 100. In the given formula, the step size of the gradient descent will change depending on whether feature value X is present in the formula. Different step sizes for each feature will result from the differences in feature ranges. Data needs to be normalized before feeding it to the model to make sure that the gradient descent progresses evenly towards the local minima and that the steps for gradient descent are updated at the same pace for all the features demonstrated in Eq 9.


$$\emptyset_{j}=\emptyset_{j}-\alpha \frac{1}{m}\sum_{i=1}^{n}(h_{\theta }(x^{\left(i\right)})-y^{\left(i\right)})x_{j}^{\left(i\right)}$$ Eq 9

For the trained classification algorithm to work properly, the primary data must first undergo some sort of data normalization due to the high level of irregularity present in the primary data. If the data is not normalized, the model will be dominated by variables on a larger scale, which will have a detrimental effect on the model’s efficiency. As a result, normalization is an absolute necessity. The min-max scaling method given in Eq 10 can be used to rationalize the set of diverse data.


$$X^{\prime }=\frac{X-min(x)}{max(x)-min(x)}$$ Eq 10

### 3.11. Parameter configuration

For the decision tree, the maximum number of leaf nodes is set to 200. The minimum sample size per leaf was raised to 2 to compel each leaf to collect pertinent information. Additionally, a minimal cost-complexity pruning impact was added using the ccp_alpha complexity parameter like regularization. When set to the minimum value, pruning iteratively locates the node with the "weakest connection." The weakest link is defined by its effectual alpha, with the nodes with the lowest effective alpha deleted first. The same parameter values used for the DT were evaluated for RF, with favourable results. Regarding ET, the maximum number of leaf nodes is set to 500, maximum depth of nodes is 300 with n_estimators set to 200. Table 3 shows the parameters of the tree-based algorithms.

**Table 3:** Parameters of tree-based classifiers.

Parameters	Tree Based Models			
	Decision Tree	Random Forest	Light GBM	Extra Trees
Min_samples_leaf	2	2	2	2
Max_leaf_nodes	100	100	50	500
Max_depth	30	30	30	300
Ccp_alpha	1.e-3	1.e-3	-	1.e-3
N_extimators	-	-	-	200

Multi-layer perceptron was applied to detect network attacks where the parameters included adaptive moment estimation (Adam) as an optimizer with a 0.0001 learning rate. As shown in Table 4, the parameters of the convolutional neural network included a 0.001 learning rate and an Adam optimizer. Early stopping for both models was specified to run the model 2 times more before stopping to avoid overfitting. The activation function adopted was the rectified linear activation unit, and the output was softmax. To lessen overfitting, dropout layers, and early stopping were used.

**Table 4:** DNN architectures.

Parameters	MLP	CNN
Activator	Relu, Softmax (Output)	Relu, Softmax (Output)
Optimizer	ADAM	ADAM
Learning rate	0.001	0.01
Loss	Categorical cross-entropy	Categorical cross-entropy
Hidden layers	5	3
Neurons per layer	512, 256,128, 64	128, 32,16
Batch size	128	128
Conv Layer	-	5
MaxPooling	-	2

### 3.12. Feature transferability evaluation

The main differentiation between AWID (possibly referring to AWID2) and AWID3 lies in their distinct emphases and contexts. Although both datasets pertain to Wi-Fi intrusion detection, AWID3 is specifically tailored for corporate applications of the protocol, which often entails more robust security features. The key differences can be summarized as follows:

- **Protocol Focus:**

AWID (AWID2) centers around conventional Wi-Fi intrusion detection scenarios, while AWID3 is geared towards business implementations of the protocol.

- **Security Measures:**

AWID3 considers the incorporation of enhanced security measures that are prevalent in business settings. This includes the utilization of Protected Management Frames (PMF), introduced with the 802.11w amendment, which enhances Wi-Fi network security.

- **Network Architecture:**

AWID3 considers a wide range of network architectures commonly observed in commercial organizations. Consequently, the dataset encompasses information from more intricate network configurations unique to business Wi-Fi deployments.

Conclusively, while both AWID (AWID2) and AWID3 are pertinent to Wi-Fi intrusion detection, AWID3 provides a more specialized and focused dataset tailored for detecting intrusions in enterprise Wi-Fi environments. Its emphasis on better security mechanisms and diverse network designs enhances its relevance for real-world applications in the corporate sector.

The primary objective of testing feature transferability is to identify the most robust and advantageous features that can be effectively applied and generalized across diverse network environments, especially between a general Wi-Fi network setting and an enterprise network setting. This research aims to uncover features that retain their effectiveness when transferred from a general Wi-Fi network environment to a corporate Wi-Fi network environment, with a specific focus on AWID3, which pertains to enterprise versions of the protocol. The incorporation of stronger security measures and varying network topologies in the corporate context may necessitate the utilization of specific features for efficient intrusion detection.

Both datasets share common instances of deauthentication attacks. The training set encompasses AWID2-CLS-R, containing only the Normal and Flooding classes, while the test set comprises AWID3 Deauth. pcap, featuring solely the Normal and Deauthentication traffic.

## 4. Experiments and results discussion

This section discusses the feature selection and classification process. The model was created on Google Colab with T4 GPU using the open-source TensorFlow Keras framework. Data preprocessing was performed to remove any inconsistencies. This consisted of handling missing information, standardizing data formats, and implementing the required transformations to ensure consistency and correctness. Machine learning models were trained and assessed. This entailed dividing the dataset into training and testing subsets, employing cross-validation techniques, and utilizing suitable evaluation metrics to measure model performance. The ethical aspects of implementing automated wireless intrusion detection methods were addressed. The data collected and analysed by these technologies was solely utilised to improve network security and combat cyber-attacks. Ethical considerations require that the data be treated responsibly and ethically, with strict measures in place to protect sensitive information from unauthorised access or misuse. By following these ethical standards, it was assured that automated intrusion detection systems positively contribute to network security while respecting individual privacy rights and sustaining faith in technology. The AWID3 dataset used in these experiments has 5 classes: normal, de-authentication, disassociation, Krack, and Kr00k. Intrusion detection datasets are generally highly imbalanced as attack traffic is always significantly less than normal traffic. In this case, balancing the data with the use of oversampling techniques would not be appropriate. In this approach, stratified k-fold with 10-fold cross-validation (CV) has been implemented to neutralize the imbalance characteristic of the dataset.

### 4.1. Evaluation metrics

The following are the appropriate evaluation metrics to detect cyberattacks. The efficiency of the proposed methodology has been evaluated using a confusion matrix that consists of true-positive (TP), true-negative (TN), false-positive (FP), and false-negative (FN) as defined below:

- True-positive (TP): number of instances successfully categorized as cyber-attacks.
- True-negative (TN): number of instances that are categorized as normal/regular network traffic.
- False-positive (FP): number of instances wrongly categorized as any cyber-attack.
- False-negative (FN): number of instances of cyberattacks remained undetected by IDS.

Accuracy, Precision, Recall, and F1 measures have all been used in this study as assessment metrics based on the characteristics of the confusion matrix.

**Accuracy:** The ratio of cases in the dataset that the model correctly identified. The higher the accuracy, the better the model applied.


$$Accuracy=\frac{True_{Positve}+True_{Negative}}{True_{Positive}+True_{Negative}+False_{Positive}+False_{Negative}}$$

**Precision:** The ratio of the number of true positive instances that are classified exactly to the total number of positive instances (true positive and false positive).


$$Precision=\frac{True_{Positive}}{True_{Positve}+False_{positive}}$$

**Recall:** The ratio of true positive instances that are precisely labeled as true positive to all true positive instances. This means the value of recall will be low when the FN rate is high.


$$Recall=\frac{True_{positve}}{True_{positive}+False_{positive}}$$

**F1-score:** This is referred to as the harmonic mean of the accuracy and recall metrics. It is regarded as a useful assessment criterion for unbalanced data.


$$f1-score=\frac{2True_{positve}}{2True_{positive}+False_{positive}+False_{Negative}}$$

### 4.2. Feature selection and classification for all attacks

The process of feature selection presented various limitations throughout the investigation. One notable limitation revolved around the potential existence of irrelevant or redundant features within the dataset. The extensive features, while furnishing information, introduced complexities in discerning the most pertinent ones. Moreover, ensuring the transferability of chosen features across diverse network environments emerged as a persistent challenge. The ever-changing landscape of wireless networks compounded the intricacies of the feature selection procedure. Despite the application of rigorous methodologies, the ongoing struggle to strike a balance between achieving a lightweight model and maintaining requisite detection accuracy remained a formidable constraint. The wrapper method ranks the feature subsets according to how well they can classify the objects using the learning machine. Fundamentally, recursive feature elimination prioritizes features based on a relevance metric. RFE acts as a greedy algorithm and strategically performs feature ranking by prioritizing features by recursively finding the reliant collinear features while removing the weak features. In this approach, RFE with a decision tree (DT-RFE) has been implemented and the eight most relevant features have been extracted that will be ideal for building a cost-effective, lightweight IDS, reducing the dimensions of data. DT-RFE can categorize powerful predictors of a given outcome without assuming the model’s internal mechanism. As mentioned above, DT-RFE selects the most meaningful features based on their ranks. Table 5 shows the most relevant selected features.

**Table 5:** DT-RFE features.

Selected Features	
1	frame.len
2.	radiotap.channel.flags.cck
3.	radiotap.channel.freq
4.	radiotap.dbm_antsignal
5.	wlan.duration
6	wlan.fc.retry
7.	wlan.fc.subtype
8.	wlan.fc.type

**Fig 4:**
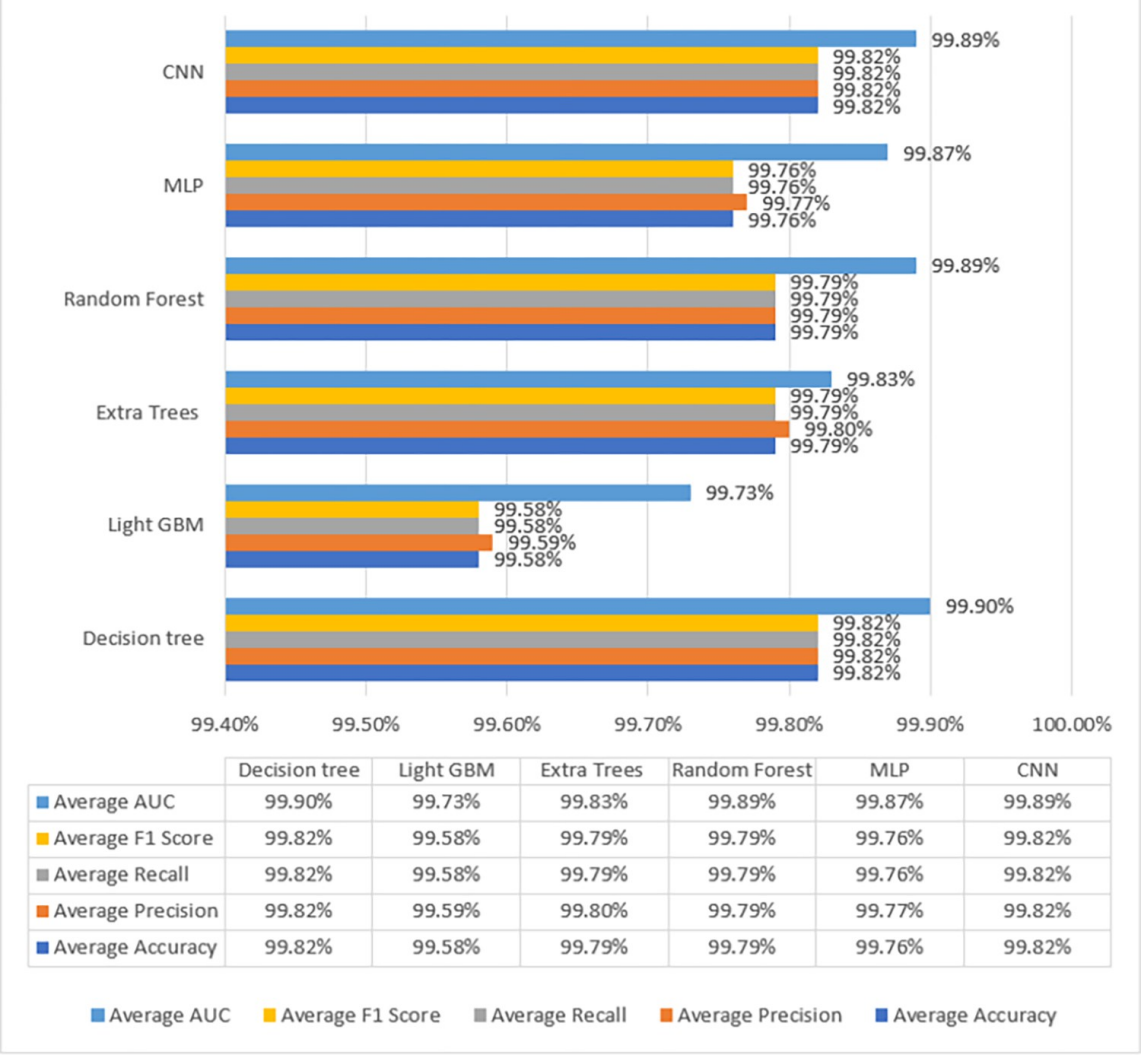
Performance evaluation.

Apart from these 8 features, another feature wlan_radio.signal_dbm, which represents the broadcasting device’s signal strength, was used for classification. When used with the radiotap.dbm antsignal, it can help pinpoint flooding and impersonation attacks. The use of this feature in combination with the other features reduced the false-positive rate. Certainly, "wlan_radio.signal_dbm" is essential to the cyberattack detection feature selection process. This feature represents a wireless network’s signal strength and provides important information about the reliability and quality of the Wi-Fi connection. When "wlan_radio.signal_dbm" was incorporated along with a subset of eight other relevant attributes, the number of false positives during cyberattack detection was significantly reduced. This decrease suggests that by providing insightful contextual information about the wireless network environment, "wlan_radio.signal_dbm" enhances the selected subset of attributes. This feature integration improves the intrusion detection system’s overall accuracy and effectiveness by providing more insights into network behavior and possible security threats. The classification results are shown later in Fig 4. Since the dataset is imbalanced, the results should be considered in terms of the F1-score and area under the curve (AUC) score.

From Table 5, it is observed that CNN and DT have attained the best results in terms of accuracy, precision, recall, F1-score, and AUC score. However, the decision tree attained slightly better results in terms of F1-score, AUC score, and processing time of 99.82%, 99.90%, and 20.2s respectively. Decision Tree also attained the highest recall value of 99.82%. A high recall value is essential in wireless networks. If an attack instance is incorrectly classified as regular network traffic, it can cause a major loss of data in real-world businesses.

**Fig 5:**
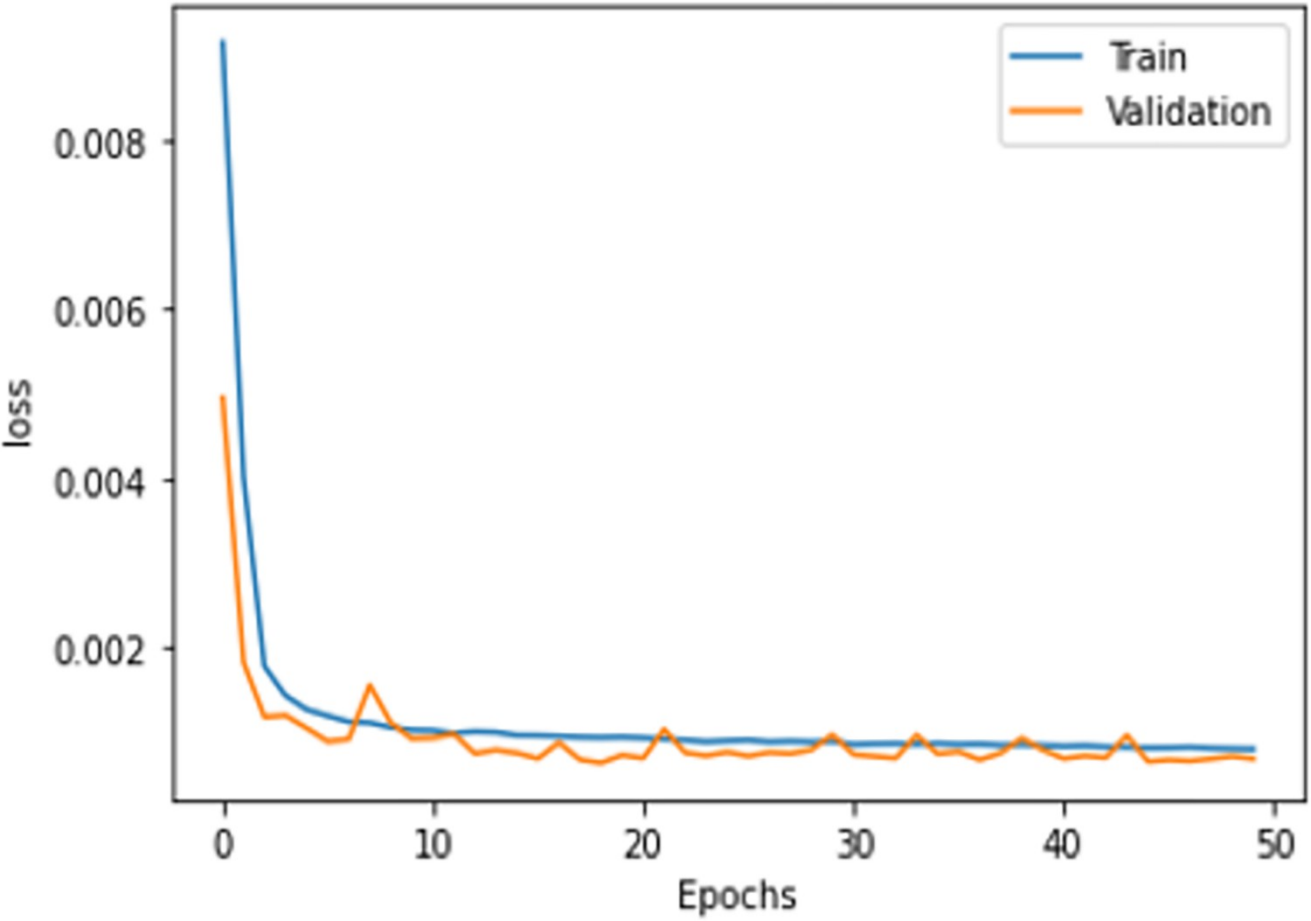
MLP.

**Fig 6:**
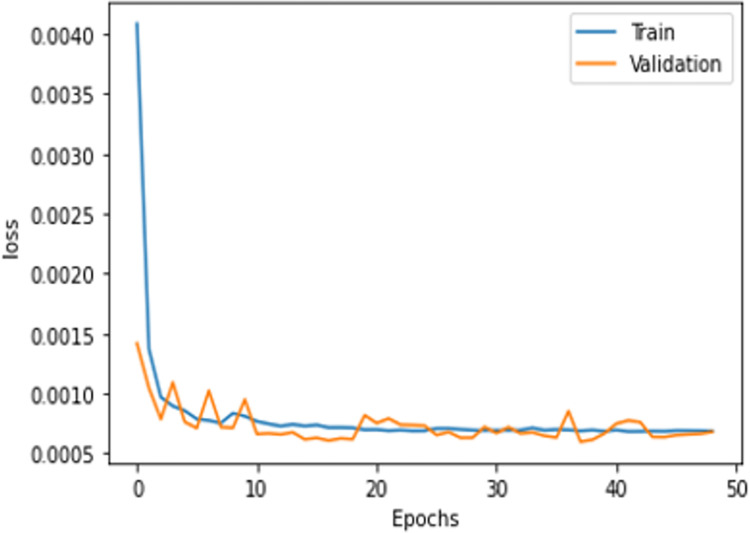
CNN.

Regarding deep learning architectures, the validation loss is less than the training because of the dropout layers used in the model [4]. Figs 5 and 6 show the average loss among all folds. To train the MLP and CNN architectures, it was found that the number of samples was insufficient. The ideal loss value was easily obtained by these architectures within four to five epochs. The models were therefore trained with quite a small loss, with the loss decreasing by a trivial 0.001 after each epoch. Summing up the results, machine learning models can be used to train data for an economical and time-saving cyber-attack detection mechanism. That would be suitable for small and medium enterprises (SMEs) as well. However, for large-scale data, DNN models are preferred.

**Fig 7:**
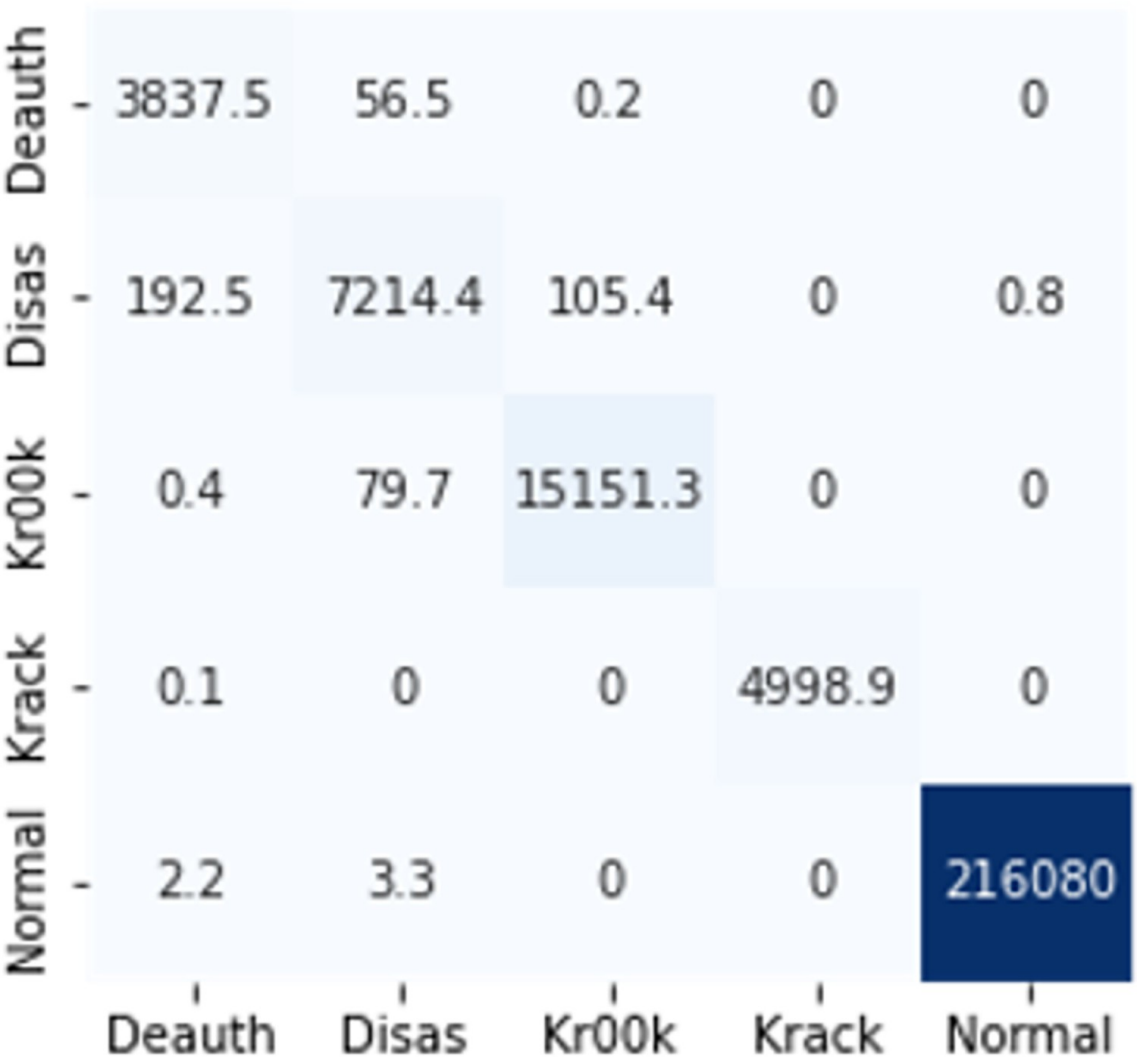
Decision tree.

**Fig 8:**
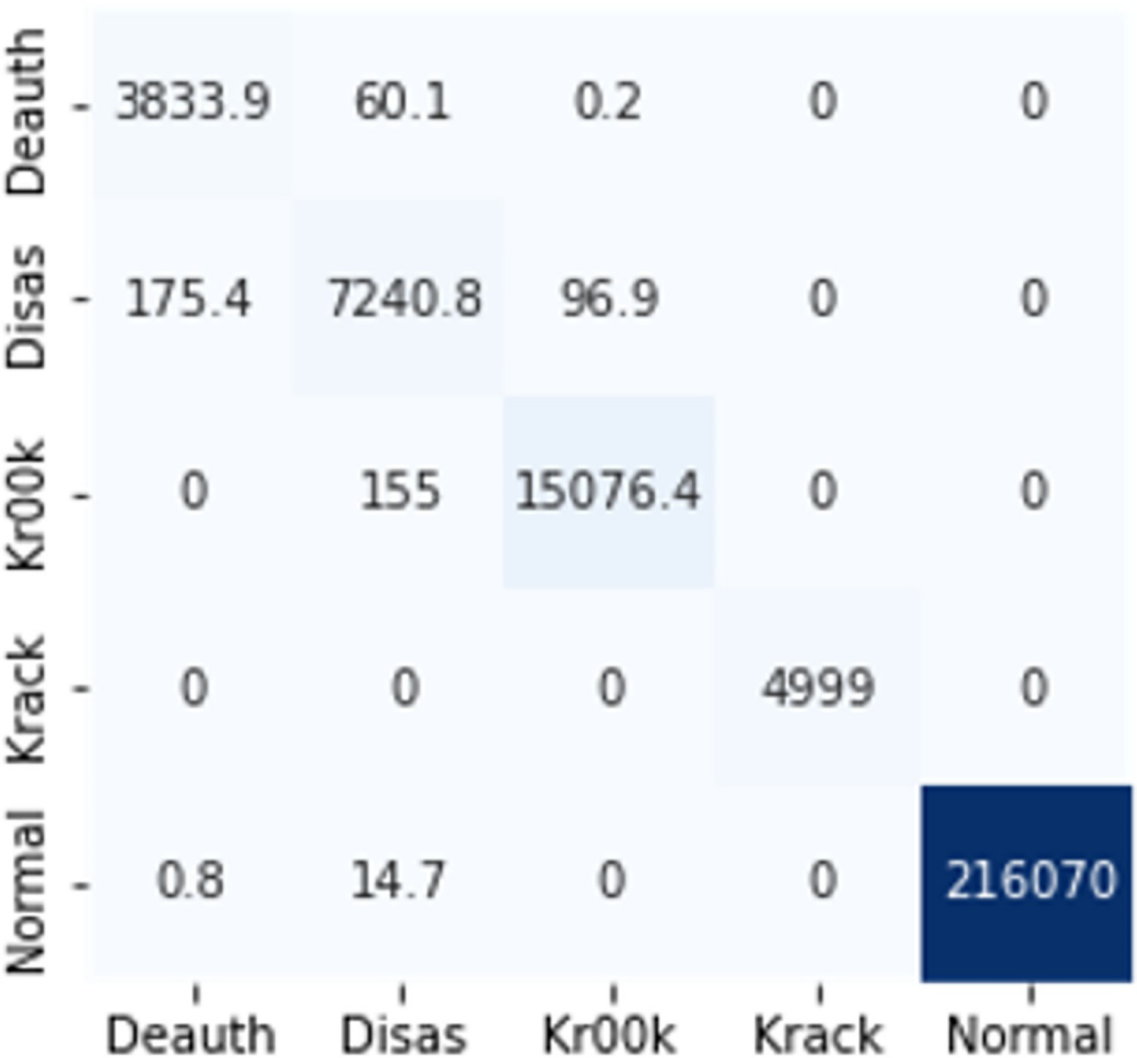
Random forest.

**Fig 9:**
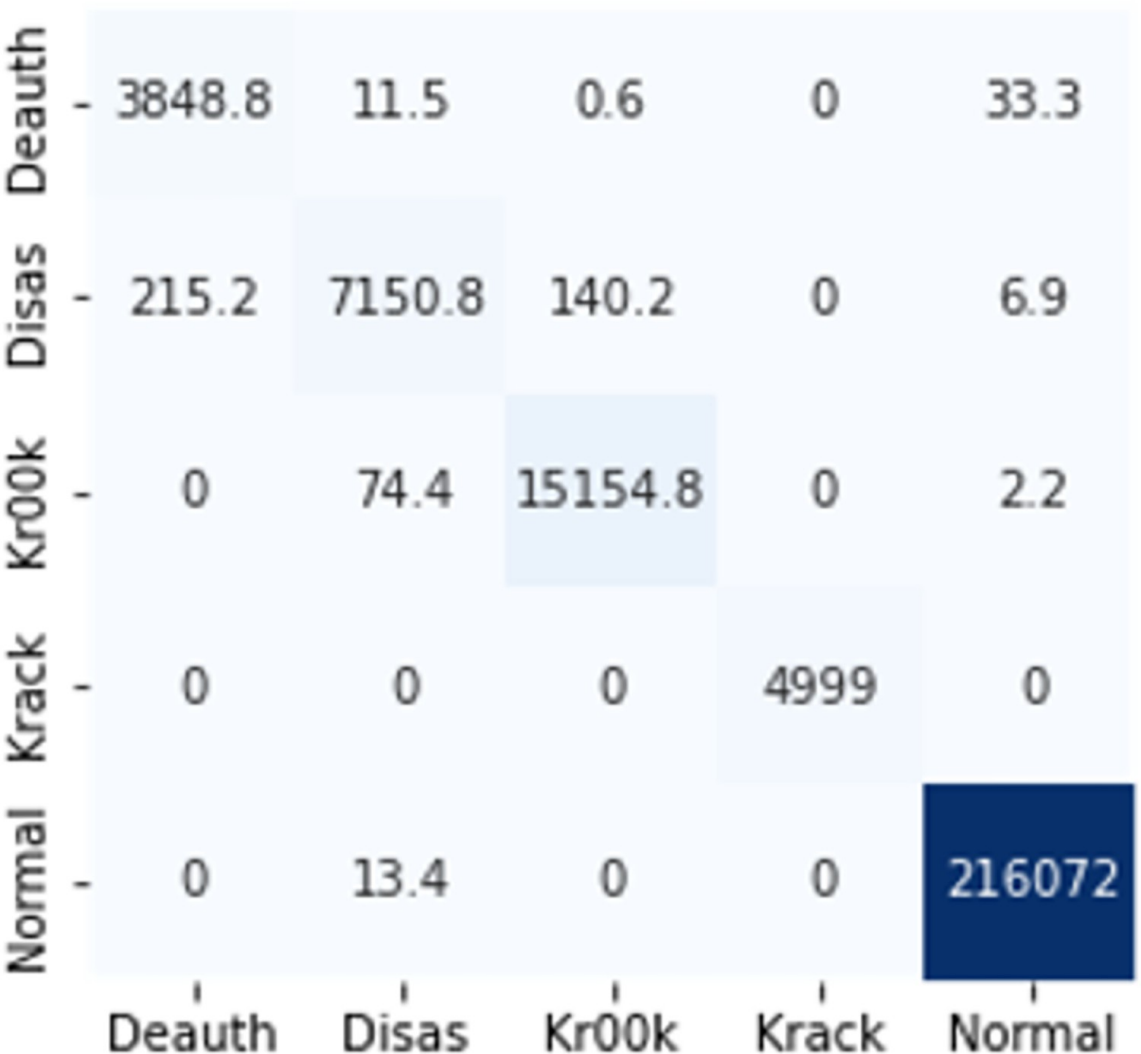
Extra trees.

**Fig 10:**
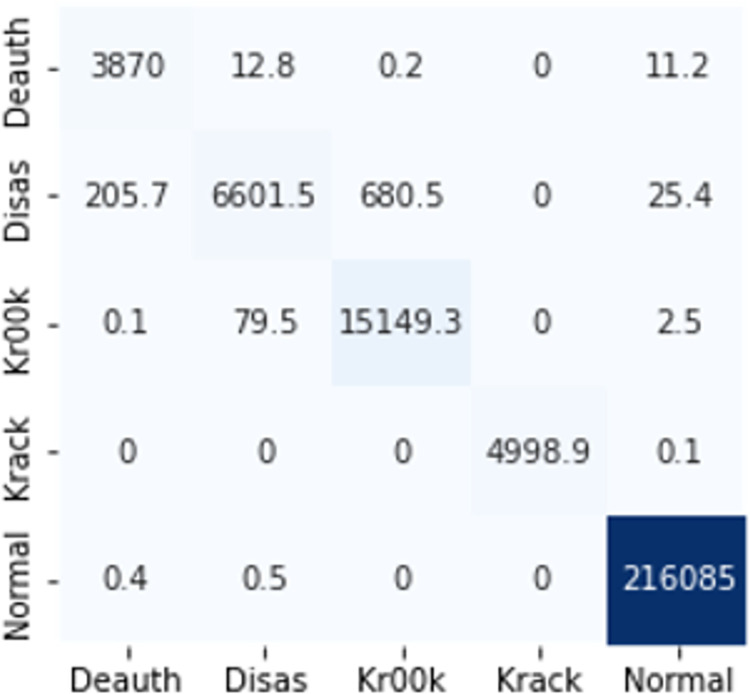
Light GBM.

**Fig 11:**
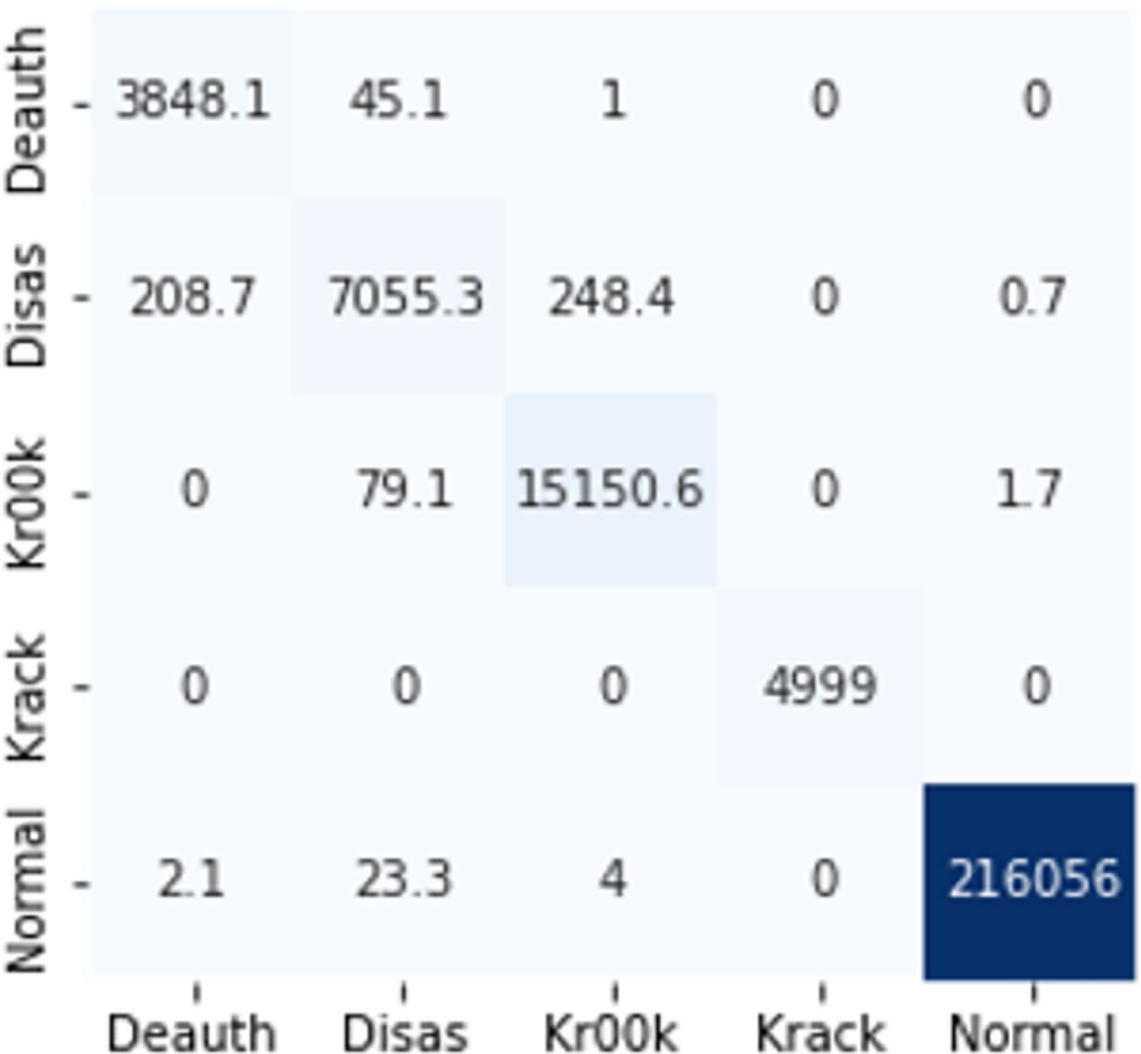
MLP.

**Fig 12:**
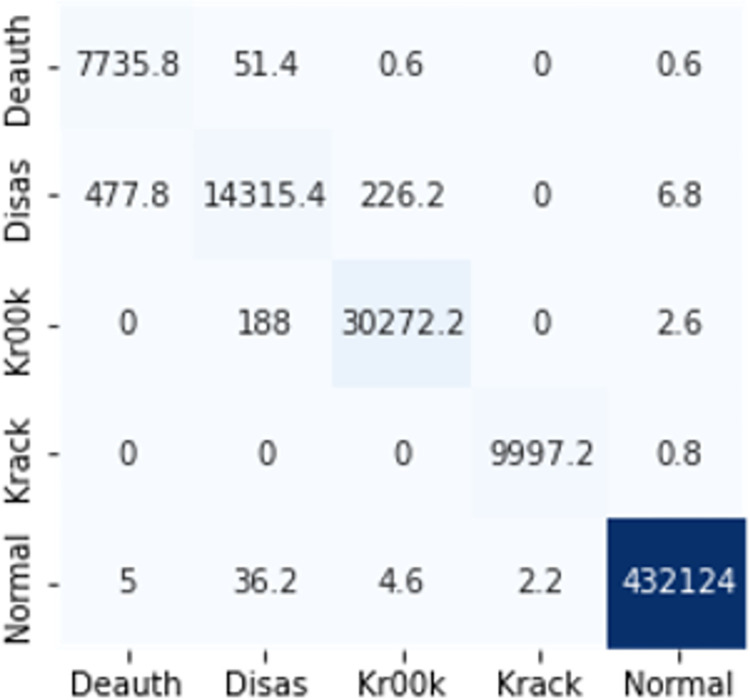
CNN.

**Fig 13:**
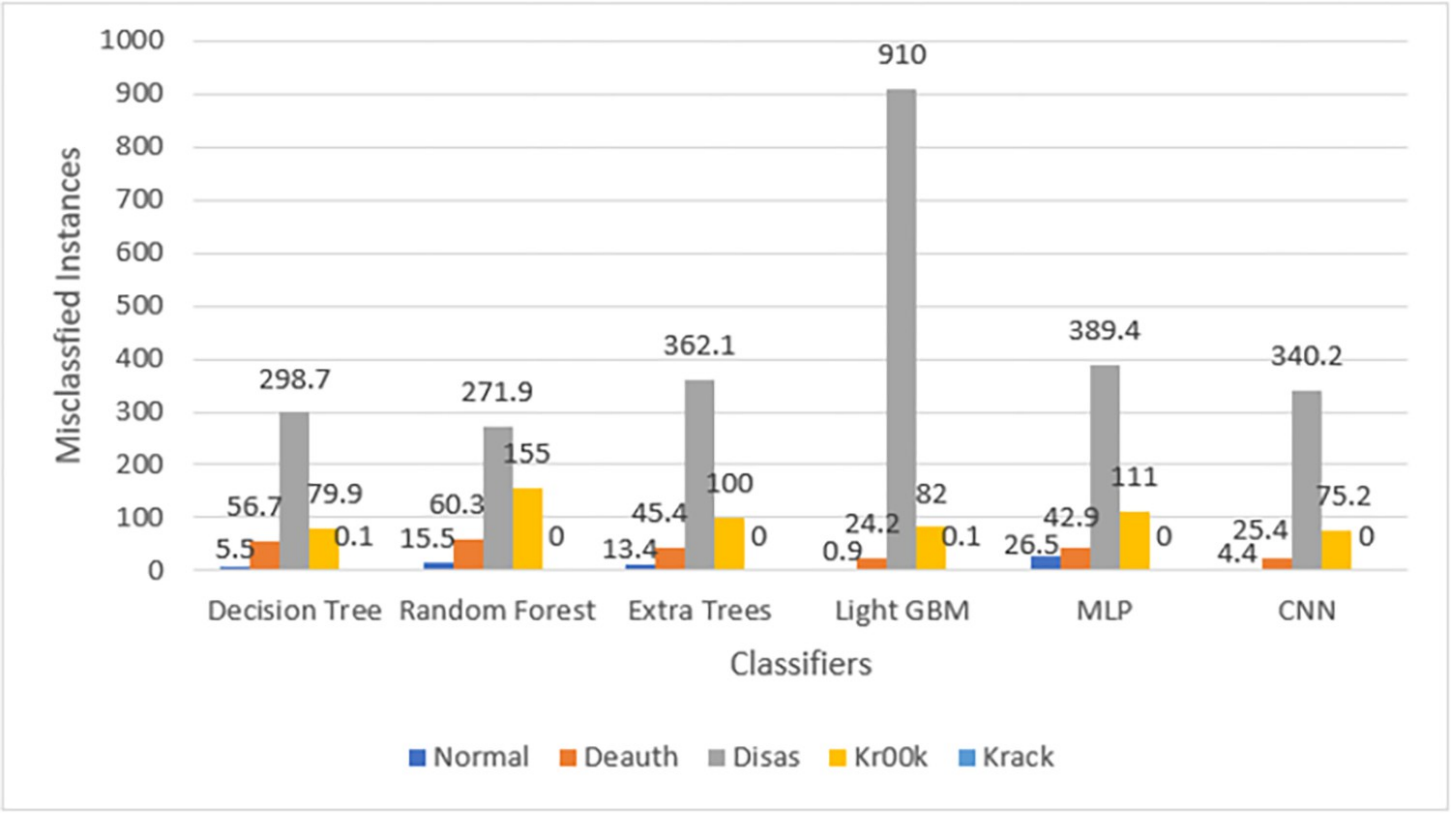
Average number of misclassified instances.

The confusion matrices in Figs 7–12 demonstrate the average confusion matrices of classifier analysis. Normal, de-authentication, and Krack attacks demonstrated significant accuracy as less than 100 instances are falsely classified with tree-based models. Whereas Light GBM and MLP classifiers had a hard time differentiating between Kr00k and disassociation attacks. Approximately 400 instances of these attacks were falsely classified even with 16 features set. Fig 13 shows that DT and CNN have the least number of misclassified instances.

These results demonstrate that the DT-RFE method can reduce features from 16 to 8 for the detection of these attacks using DT. This could be helpful for attack detection in an enterprise network. With 8 core features and an additional feature out of 16, the proposed method can detect Wi-Fi cyber-attack with little processing time and a high detection rate.

Furthermore, stratified cross-validation proved efficient to alleviate the effects of overfitting and class imbalance. Despite being under-presented de-authentication and Krack classes with only 38,942 and 49,990 samples, the detection rate is quite high.

### 4.3. Feature transferability

**Fig 14:**
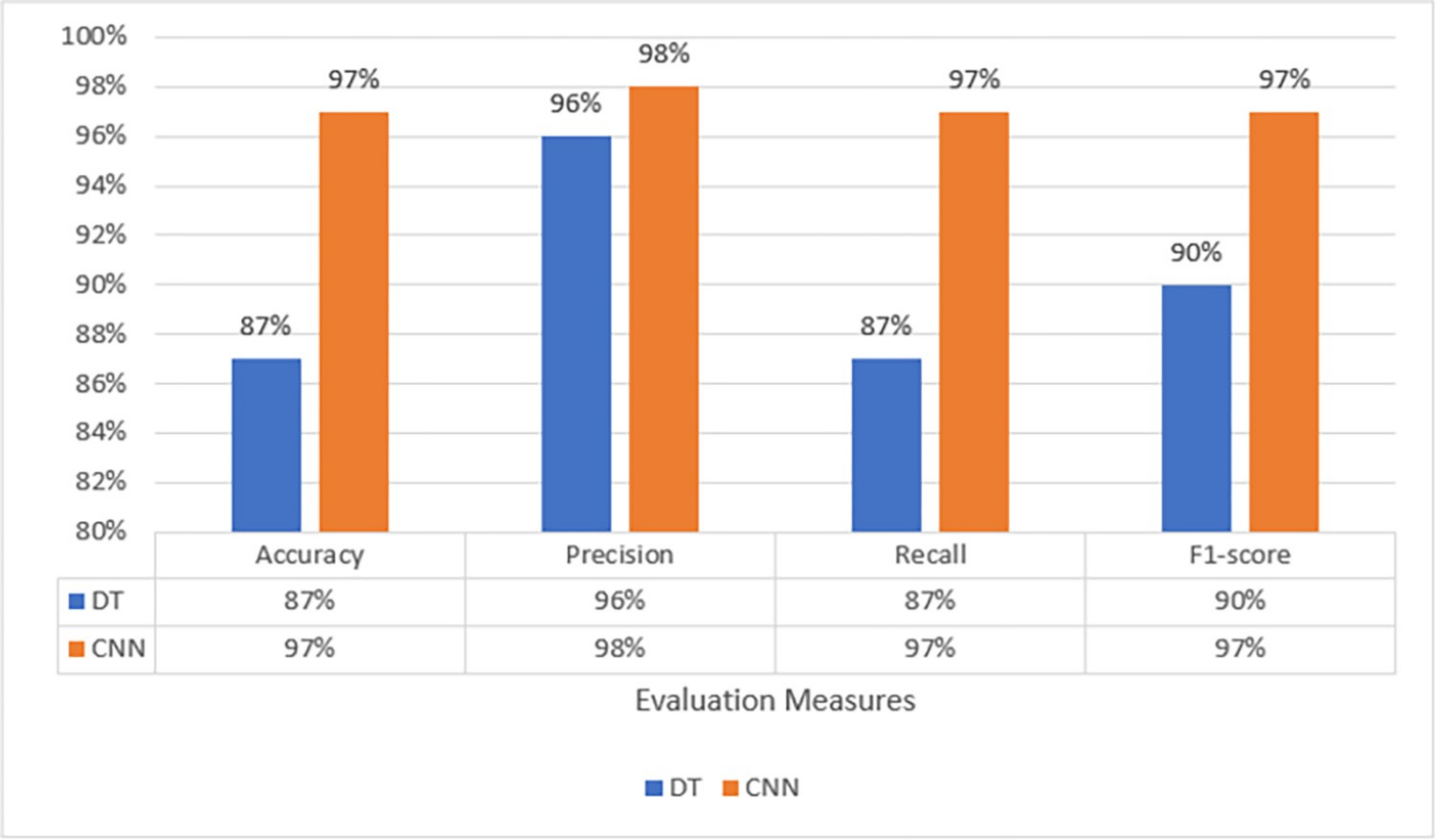
Feature transferability evaluation.

According to the analysis done by [4], 30 features and 27 features were not transferable whereas 13 feature sets and 5 feature sets were transferable, but the results attained needed to be improved. For this purpose, radiotap.channel.flags.cck and radiotap.dbm_antsignal were excluded to better comprehend this result. Radiotap.channel.freq, radiotap.flags.type.cck, and other features fall under this category. The fact that AWID2 and AWID3 were recorded on several radio stations had an impact on this decision. Furthermore, neither of the two flag-based features provided insightful information for identifying flooding assaults. Only the best models from ML and DL techniques such as DT and CNN have been used to evaluate the transferability of features and the evaluation of the results is given in Fig 14.

**Fig 15:**
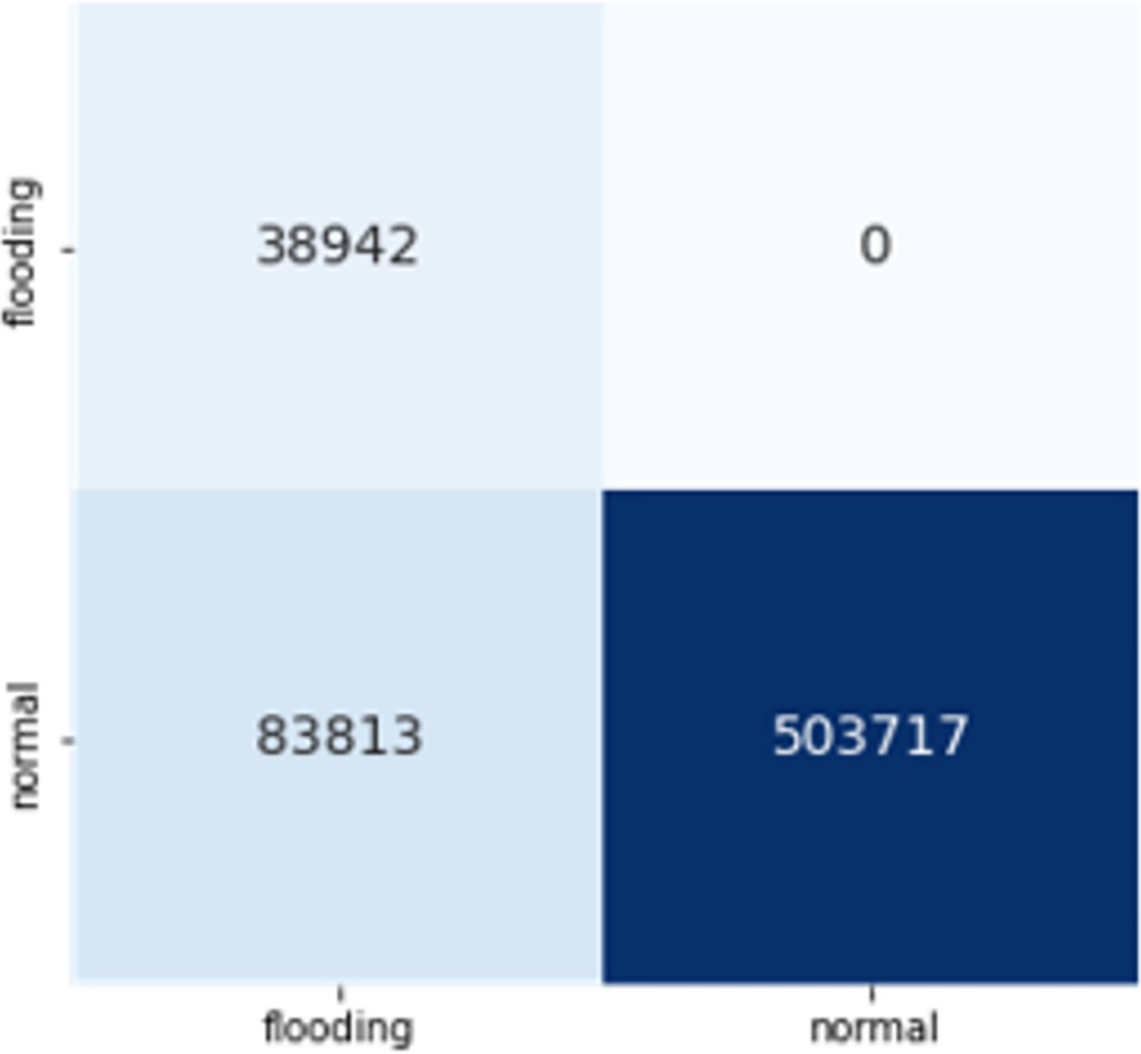
Transferability with DT.

**Fig 16:**
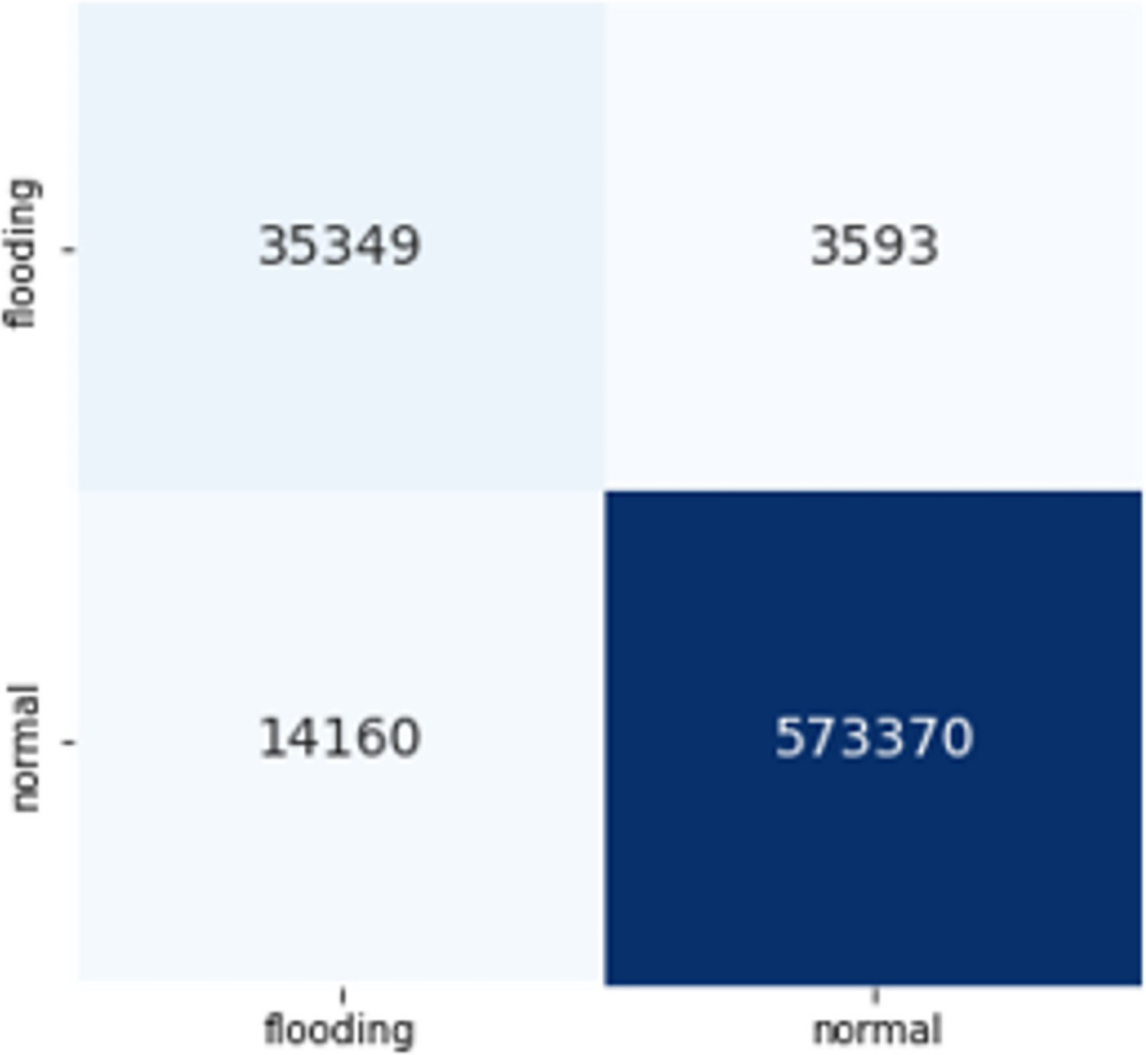
Transferability with CNN.

The confusion matrices of both DT and CNN with these features are given in Figs 15 and 16. The Decision Tree (DT) model shows zero instances of normal traffic being misclassified as flooding, resulting in no false positives. However, there is a significant issue with false negatives, where around 84,000 instances of deauthentication attacks are wrongly classified as normal. On the other hand, the Convolutional Neural Network (CNN) exhibits notably low numbers of both false positives and false negatives, making it a superior choice for feature transferability.

CNN’s superior performance in accuracy, precision, recall, and F1-score indicate its effectiveness in handling the classification task compared to DT. These results suggest that for this specific problem, CNN is a more suitable choice, as it provides a higher overall predictive capability with better precision and recall. The results demonstrated that these 6 features are transferable, achieving 90% and 97% F1 scores respectively.

### 4.4. Recursive feature elimination for reduced feature set

DT-RFE was applied on 4 separate datasets (de-authentication/normal, disassociation/normal, Krack/normal, Kr00k/normal). A different subset of features has been extracted with three of the most useful features out of the mentioned 16 features. A stratified cross-validation score equal to 5-fold is used to avoid over-fitting and handle data imbalance problems. For each attack, different subsets of features are identified. These feature subsets could classify the incoming cyberattacks based on 3 top ranking features. Table 6 shows the combination of features for each attack.

**Table 6:** Features for each attack.

Deauthentication	Disassociation	Krack	Kr00k
’frame.len’, ’wlan.fc.type’, ’wlan.duration’	’frame.len’, ’wlan.fc.type’, ’wlan.duration’	’radiotap. channel. freq’, ’wlan. duration’, ’wlan.fc. protected’	’frame.len’, ’wlan.fc.type’, ’wlan.fc.subtype’

Table 7 demonstrates the performance of algorithms on each attack. Three classifiers with the best performance including DT, RF, and ET were used for testing the AWID3 dataset. Random forest attained superficial results with maximum AUC and F1 scores for each attack. The decision tree completed the analysis in 2 seconds for de-authentication attacks. However, for the rest of the attacks, the decision tree was prone to overfitting.

**Table 7:** Performance metrics for feature reduction of each attack.

Model	Accuracy	Precision	Recall	F1 score	AUC score	Execution time
**Deauthentication Attacks**						
Random forest	99.99%	99.99%	99.99%	99.99%	99.99%	1min 50s
Extra trees	99.33%	99.40%	99.33%	99.35%	99.64%	1min 9s
Decision Tree	99.18%	99.28%	99.18%	99.21%	99.56%	2.2s
**Disassociation Attacks**						
Random forest	99.72%	99.73%	99.72%	99.72%	99.83%	57.7s
Extra trees	99.92%	99.92%	99.92%	99.92%	99.93%	49.5 s
**Krack Attacks**						
Random Forest	99.17%	99.18%	99.17%	99.16%	99.43%	27.5 s
Extra Trees	99.88%	99.89%	99.88%	99.88%	99.78%	23 s
**Kr00k Attacks**						
Random Forest	99.91%	99.91%	99.91%	99.91%	99.95%	3min 44s
Extra Trees	99.81%	99.92%	99.81%	99.81%	99.88%	3min 18s

The confusion matrices in Figs 17–25 show the average results of all 10 folds for machine learning analysis. It is observed that with ET, approximately, only 150 instances were misclassified.

**Fig 17:**
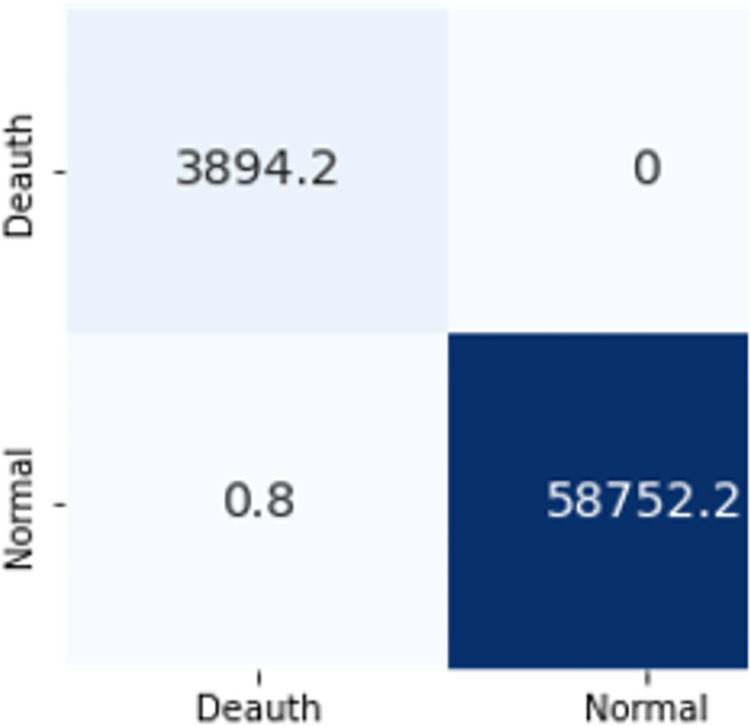
Random forest.

**Fig 18:**
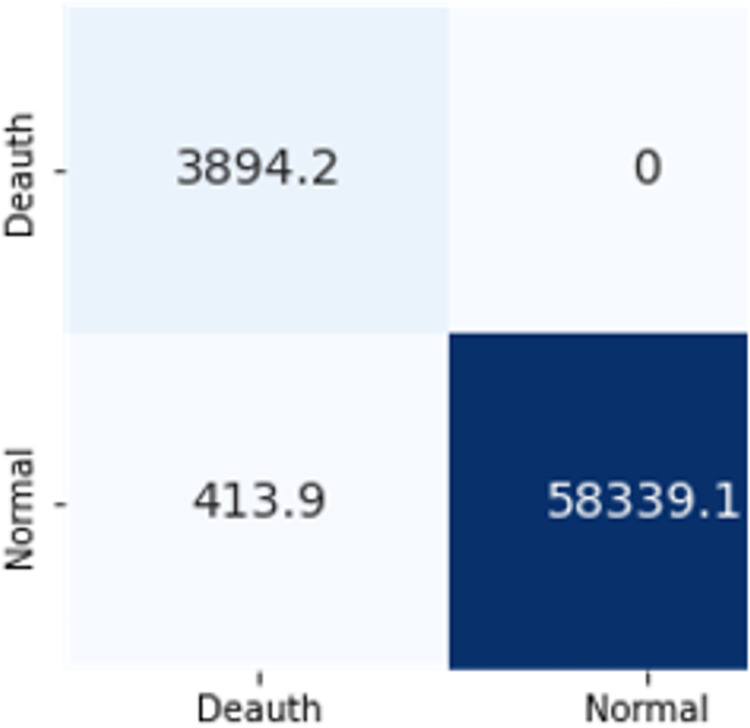
Extra trees.

**Fig 19:**
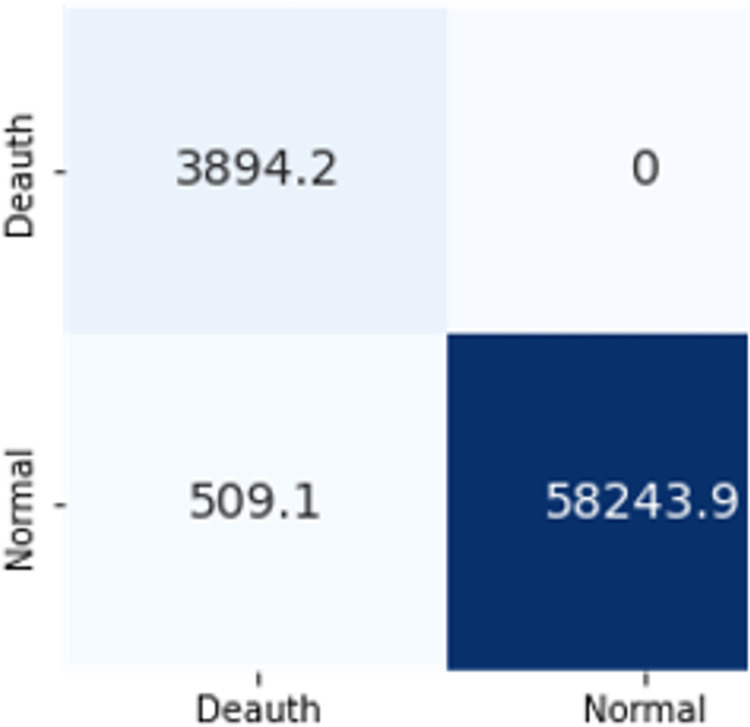
Decision trees.

**Fig 20:**
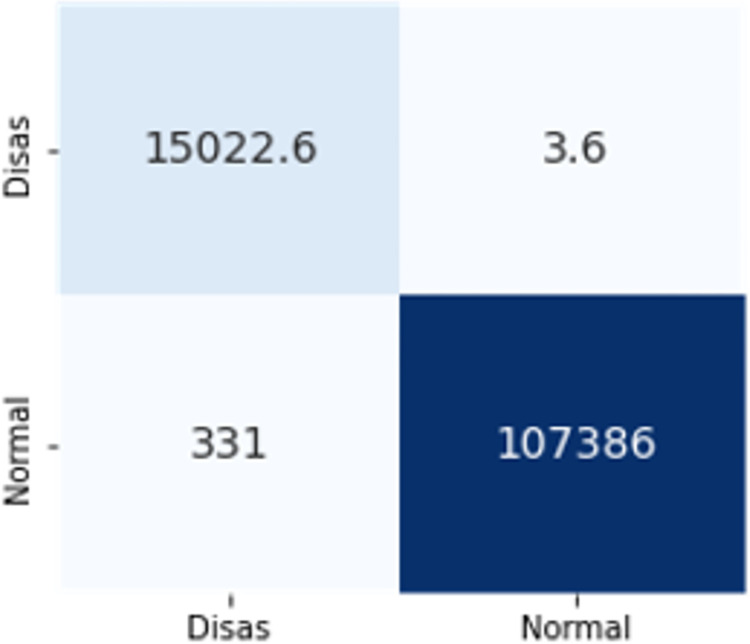
Random forest.

**Fig 21:**
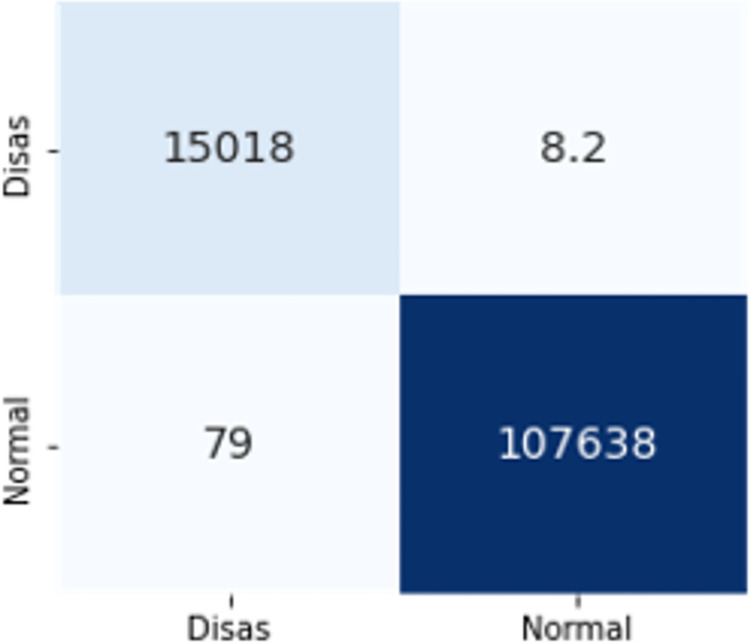
Extra trees.

**Fig 22:**
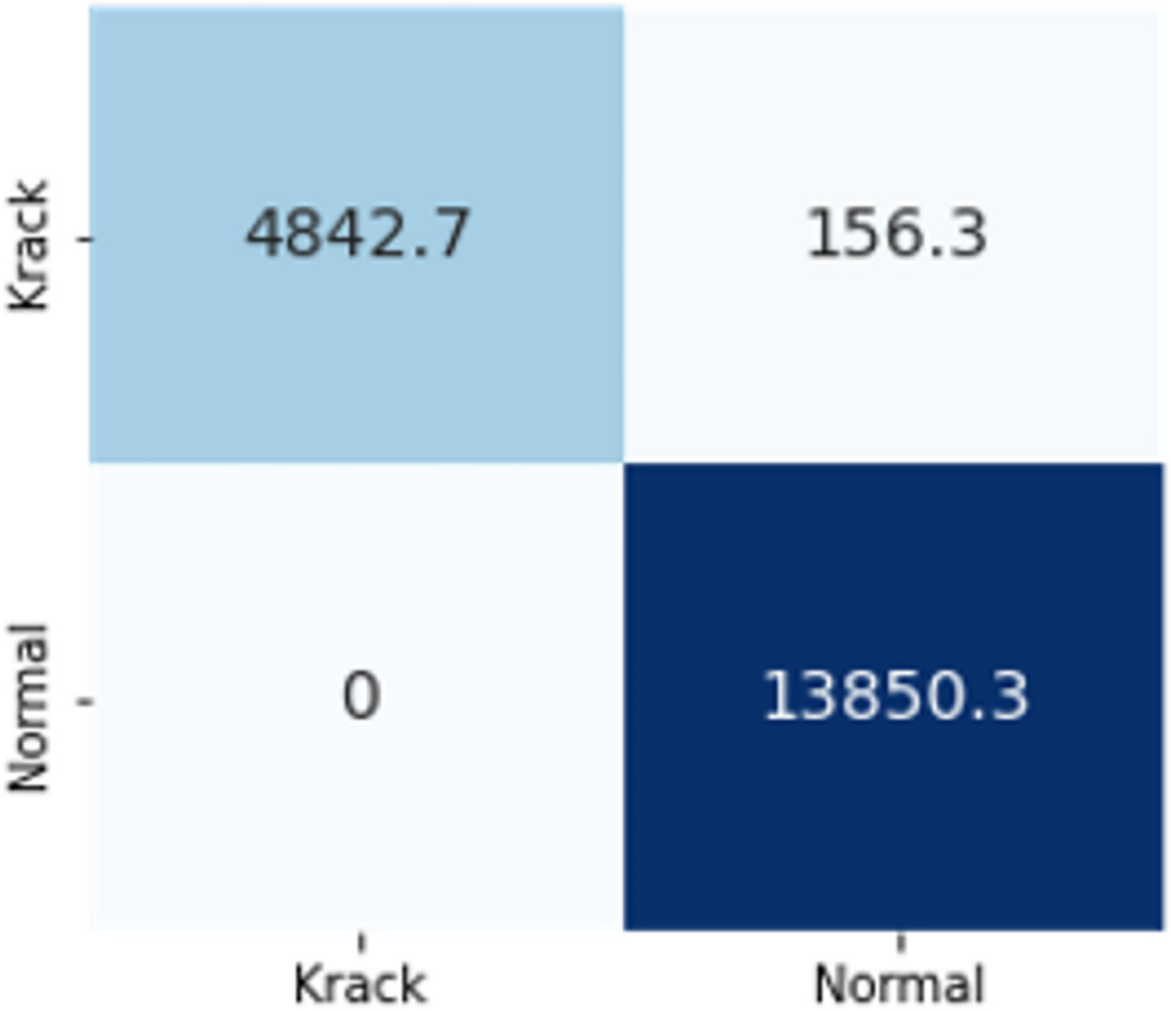
Random forest.

**Fig 23:**
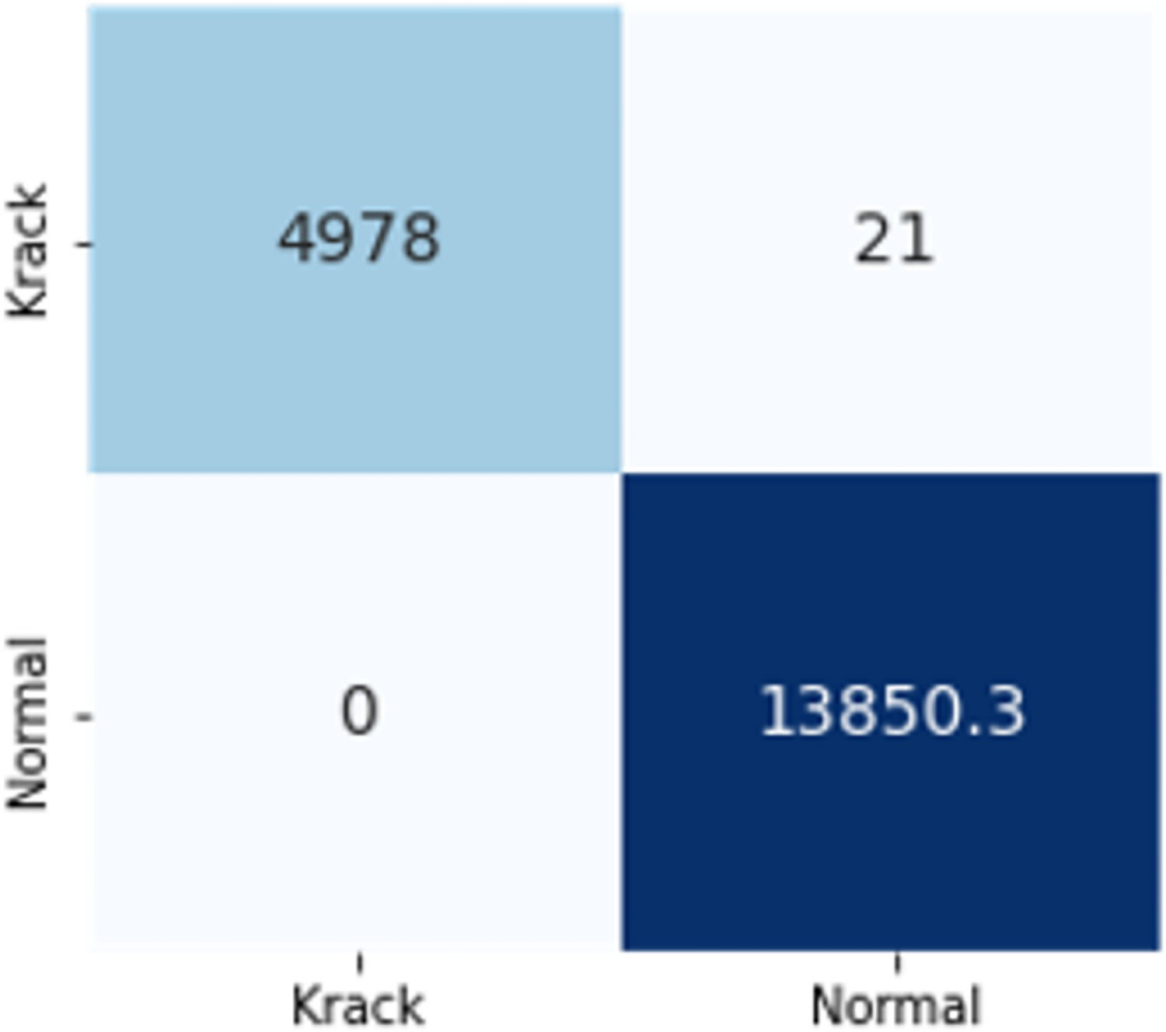
Extra trees.

**Fig 24:**
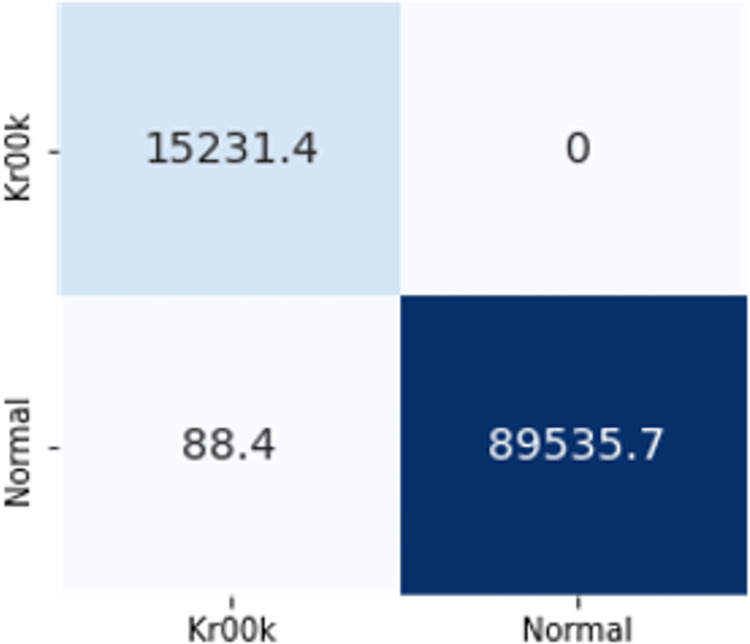
Random forest.

**Fig 25:**
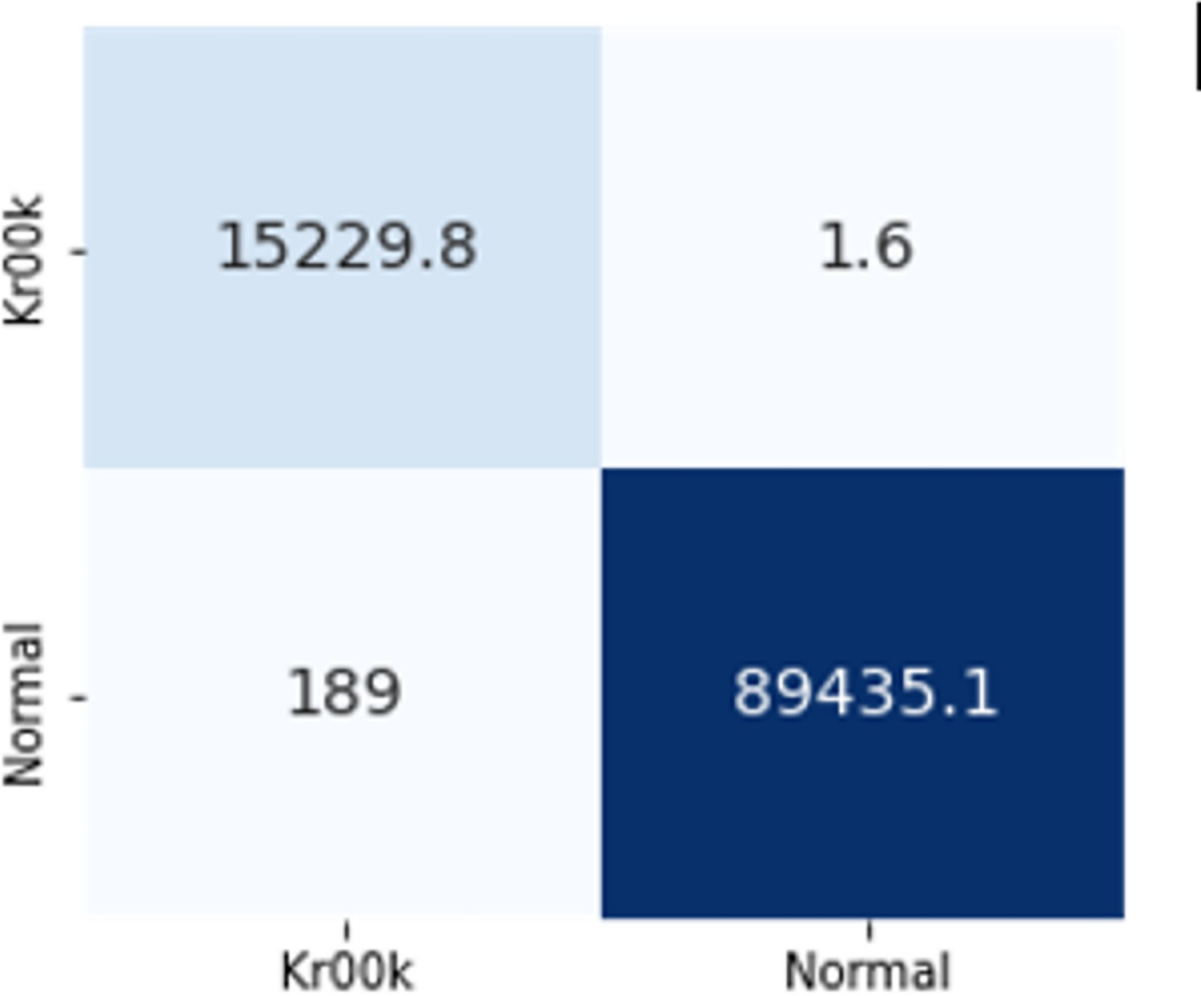
Extra trees.

It is simpler to understand the model’s results when a more condensed collection of features is used. You can more easily see the significance of each aspect in classifying various attacks. The modelling process can be streamlined, model performance can be improved, and a better knowledge of the essential features driving attack classification can be obtained by choosing distinct feature subsets for classifying cyberattacks depending on the top-ranking features. When working with complicated, high-dimensional datasets, it is a useful strategy.

### 4.5. Feature generalization

Generalization refers to the capability of the classification model to adapt to previously unseen data. In this experiment, the attack classification is performed to evaluate the generalization of the extracted features of each attack. The reduced feature subsets for de-authentication and disassociation attacks were used to perform analysis on the AWID dataset.

Table 8 demonstrates the performance of feature generalization on the AWID dataset. AWID consists of two attacks from the AWID3 dataset which are de-authentication and disassociation attacks. The three most relevant features of each attack from AWID3 were tested on the AWID dataset with tree-based models. For de-authentication attacks, RF, DT, and ET while for disassociation attacks, RF and ET were utilized.

**Table 8:** Feature generalization using AWID dataset.

Model	Accuracy	Precision	Recall	F1 score	AUC score	Execution Time
**Deauthentication Attack**						
Random Forest	96.66%	96.86%	96.66%	96.65%	96.48%	5.77s
Extra Trees	96.76%	96.53%	96.76%	96.75%	96.59%	2 s
Decision Tree	96.66%	96.87%	96.66%	96.65%	96.49%	111 ms
**Disassociation Attacks**						
Random Forest	99.99%	99.99%	99.99%	99.99%	99.99	7 min
Extra Trees	99.99%	99.99%	99.99%	99.99%	99.99	3 min

The results confirm the feasibility of feature generalization and demonstrate that the extracted features from each type of attack can be effectively applied to another dataset with different network conditions for attack classification.

### 4.6. Comparison with state-of-the-art techniques

The proposed models exhibit impressive resilience against several cyberattacks, such as deauthentication, Krack, and Kr00k. These models have demonstrated the ability to efficiently identify and counteract these types of attacks, protecting wireless networks’ security and integrity, through extensive testing and assessment. Even in the midst of the complexity of real-world network environments, these models can recognize patterns and abnormalities indicative of these particular attacks by utilizing significant machine learning and deep learning approaches. This robustness highlights the dependability and effectiveness of the proposed approach in defending against a variety of cyber-attacks, offering enhanced security to both network administrators. Since the imbalanced nature of data is maintained, the F1-score should be considered rather than accuracy for evaluation. The execution time of the proposed methods was less than the previous state-of-the-art techniques. Table 9 compares the proposed work performance measures with state-of-the-art techniques. Only the weighted values of the evaluation measures were considered.

**Table 9:** Comparison with state-of-the-art techniques.

Model	Features	Classes	Accuracy	Precision	Recall	F1 score	AUC	Balanced
LightGBM [35]	5	3	82.57	82.33	82.57	82.18		-
ET [4]	16	3	99.96	99.75	99.28	99.52	99.49%	-
MLP [4]	16	3	99.73	99.65	95.68	97.55	96.47%	
kNN [48]	30	2	99%	-	-	-		✓
**Proposed work (DT)**	**8**	**5**	**99.82%**	**99.82%**	**99.82%**	**99.82%**	**99.90%**	**-**
**Proposed work (CNN)**	**8**	**5**	**99.82%**	**99.82%**	**99.82%**	**99.82%**	**99.89%**	**-**

Table 10 presents the outcomes of various models in terms of their performance metrics, including accuracy, precision, recall, and F1 score. To determine if the features could be applied to various network situations, these metrics were assessed using various sets of features. The results suggest that the proposed CNN model achieved a notable level of accuracy and well-balanced performance across precision, recall, and F1 scores, even with a smaller set of features. Furthermore, the performance of the Decision Tree (DT) models varied based on the number of features used, indicating the significance of feature selection in influencing the effectiveness of the models.

**Table 10:** Transferability—state-of-the-art performance.

Model	Features	Accuracy	Precision	Recall	F1 score
DT [4]	30 & 27	97.61	48.80	49.81	49.30
DT [4]	13	99.63	98.69	93.48	95.93
DT [4]	5	99.63	98.69	93.41	95.89
**Proposed CNN**	**6**	**97**	**98**	**97**	**97**

## 5. Conclusion and future works

An adversary can access a victim’s critical details by launching a series of attacks on the network. Intelligent machine/ deep learning-based cyberattack detection mechanisms have gained popularity due to their high efficiency and automation. This study aimed at developing a Wi-Fi-based attack detection system. The decision tree with recursive feature elimination was used to extract the most meaningful features for a cost-effective, lightweight, and time-efficient system to detect cyberattacks. However, apart from 16 features, wlan_radio.signal_dbm with eight other relevant features significantly reduced false positives. Different tree-based algorithms, such as decision tree, random forest, Light GBM, extra trees, MLP, and CNN have been used to detect four types of cyber-attacks (de-authentication, disassociation, Krack, and Kr00k) from the AWID3 dataset. In terms of accuracy, precision, recall, F1 score, and AUC, both Decision Trees (DT) and Convolutional Neural Networks (CNN) appear to perform exceptionally well. They obtain 99.82% accuracy for a five-class classification issue, which is comparable to or slightly better than other state-of-the-art models such as LightGBM, Extreme Trees (ET), and Multilayer Perceptron (MLP). The proposed approach showed that machine learning tree-based models would be appropriate for a lightweight IDS as it provides fewer computations with minimum execution time and better classification of attacks whereas MLP and CNN can be implemented for handling large and complex data. Furthermore, the evaluation of various metrics across extracted feature sets highlights the transferability of the features in diverse network contexts. The CNN model showcased impressive accuracy and a balanced performance with fewer features, while the DT models’ effectiveness varied based on feature quantity, emphasizing the crucial role of feature selection. The features for each attack were extracted using DT-RFE. The three best models DT, RF, and ET were used to evaluate the extracted features, and RF along with ET achieved excellent results across all performance metrics including accuracy, precision, recall, F1 score, and execution time for attack detection. During the evaluation, overfitting occurred with DT for disassociation, Krack, and Kr00k attacks. Conclusively, features were utilized with the AWID dataset to find out if the extracted features were generic or not. Both deauthentication and disassociation attacks in AWID were evaluated where RF and ET achieved high AUC and F1 scores. This research provides both theoretical and practical implications in the field of secure Wi-Fi communication in enterprise networks. The study’s practicality extends beyond specific datasets by taking into account the transferability of proposed features and models to various network contexts or situations. While the experimentation was carried out on benchmark Wi-Fi datasets, the absence of different benchmark datasets restricted the examination of other Wi-Fi network situations. Also, the suggested features are designed for Wi-Fi-based network setups which may limit their use in wired network settings. Despite this restriction, the study provides vital insights into Wi-Fi network security and sets the framework for future research to improve detection capabilities in a variety of network scenarios. The major shortcoming of this research is the unavailability of a proprietary dataset which is attributed to resource constraints, including budget, time, or the necessary infrastructure to collect and compile data for experimentation. For holistic security, future works can consider the development of newer datasets with attack and benign traffic from both enterprise and industrial networks and use different feature extraction and evaluation techniques for comparisons. The development of diversified datasets, exploration of different feature extraction methods, and rigorous evaluation can enhance the quality and applicability of intrusion detection systems in real-world network security. Collaboration and resource-sharing within the research community can also play a vital role in addressing these challenges.
